# Direct habitat descriptors improve the understanding of the organization of fish and macroinvertebrate communities across a large catchment

**DOI:** 10.1371/journal.pone.0274167

**Published:** 2022-09-22

**Authors:** Coline Picard, Mathieu Floury, Hanieh Seyedhashemi, Maxime Morel, Hervé Pella, Nicolas Lamouroux, Laëtitia Buisson, Florentina Moatar, Anthony Maire

**Affiliations:** 1 EDF R&D LNHE—Laboratoire National d’Hydraulique et Environnement, Chatou, France; 2 INRAE, UR RiverLy, Centre de Lyon-Villeurbanne, Villeurbanne, France; 3 Univ Lyon, Université Claude Bernard Lyon 1, CNRS, ENTPE, UMR 5023 LEHNA, Villeurbanne, France; 4 EA 6293 GéHCO, Université de Tours, Tours, France; 5 Laboratoire Écologie Fonctionnelle et Environnement, Université de Toulouse, CNRS, Toulouse INP, Université Toulouse 3 –Paul Sabatier (UPS), Toulouse, France; University of Eldoret, KENYA

## Abstract

In large-scale aquatic ecological studies, direct habitat descriptors (e.g. water temperature, hydraulics in river reaches) are often approximated by coarse-grain surrogates (e.g. air temperature, discharge respectively) since they are easier to measure or model. However, as biological variability can be very strong at the habitat scale, surrogate variables may have a limited ability to capture all of this variability, which may lead to a lesser understanding of the ecological processes or patterns of interest. In this study, we aimed to compare the capacity of direct habitat descriptors vs. surrogate environmental variables to explain the organization of fish and macroinvertebrate communities across the Loire catchment in France (10^5^ km^2^). For this purpose, we relied on high-resolution environmental data, extensive biological monitoring data (>1000 sampling stations) and multivariate analyses. Fish and macroinvertebrate abundance datasets were considered both separately and combined to assess the value of a cross-taxa approach. We found that fish and macroinvertebrate communities exhibited weak concordance in their organization and responded differently to the main ecological gradients. Such variations are probably due to fundamental differences in their life-history traits and mobility. Regardless of the biological group considered, direct habitat descriptors (water temperature and local hydraulic variables) consistently explained the organization of fish and macroinvertebrate communities better than surrogate descriptors (air temperature and river discharge). Furthermore, the organization of fish and macroinvertebrate communities was slightly better explained by the combination of direct or surrogate environmental variables when the two biological groups were considered together than when considered separately. Tied together, these results emphasize the importance of using a cross-taxa approach in association with high-resolution direct habitat variables to more accurately explain the organization of aquatic communities.

## Introduction

Freshwater ecosystems are amongst the most vulnerable and threatened ecosystems worldwide [1–3]. Developing efficient conservation plans to counteract the erosion of their biodiversity requires a better understanding of the environmental drivers and ecological processes that shape aquatic communities in space and time [4–6]. Nonetheless, characterizing organism-environment relationships in rivers is challenging because of the dendritic nature of hydrographic networks that constrains their organization [7, 8]. Historically, freshwater ecologists have attempted to measure the degree of alteration of a system by comparing its state to biological reference conditions [9, 10]. This has notably led to the formulation of river typologies and zonations to describe the natural (i.e. with a minimal degree of alteration) organization of aquatic organisms and identify their environmental determinants [e.g. 11–14]. The main environmental determinants that naturally shape aquatic communities are river thermal and hydrological regimes, topography, organic matter inputs and local (i.e. at reach scale) habitat characteristics [15–18]. Most of these drivers are structured longitudinally (e.g. increases in water temperature and discharge from the source to the mouth), forming the upstream-downstream gradient along which aquatic communities are distributed [19, 20].

A prevailing assumption states that at large scale, the different groups of freshwater organisms (e.g. fish, macroinvertebrates, aquatic plants) are assembled and structured by similar environmental filters [21], which is expected to result in shared spatial patterns [22, 23]. Consequently, most studies have focused on a single group, such as fish or macroinvertebrates, and have assumed its representativeness with respect to the other groups [6, 12, 18]. However, recent studies have questioned this assumption and investigated the spatial concordance between biological groups in freshwaters systems at different spatial scales [24, 25]. For instance, Backus-Freer and Pyron [26] showed that the spatial distribution of macroinvertebrates is better predicted by local environmental variations (e.g. in-stream cover, turbidity) than the distribution of fish, resulting in a low concordance between the organization of fish and macroinvertebrate communities. Other studies have suggested that the concordance of spatial patterns among aquatic organisms may increase with spatial scale, probably due to the weaker contribution of local specificities in coarser regional patterns [22, 23]. In addition, spatial concordance among communities may result from mechanisms other than environmental filtering, including biotic interactions, dispersal dynamics, assemblage homogenization due to the loss of sensitive taxa and scale effects [16, 23, 27]. Therefore, considering different biological groups when investigating broad ecological patterns and processes appears relevant, if not necessary, to ensure that the results are integrative and generalizable. This also allows the identification of patterns at ecosystem level, not just at population or community level.

In this study, we aimed at comparing the capacity of surrogate *vs*. direct environmental variables to explain the organization of aquatic communities in a large catchment. Surrogate variables refer to variables classically used as indirect proxies for the environmental conditions experienced by aquatic organisms (e.g. air temperature to approximate water temperature) or that measure general characteristics of the river reach not directly related to the habitat of the organisms (e.g. discharge to approximate current velocity or water depth) [28]. Many studies have had to rely on surrogate variables in the absence of finer or more precise data available at large scale or over a sufficiently long period [e.g. 5, 29–31]. In contrast, direct variables are those that directly translate the environmental conditions experienced by aquatic organisms. For this purpose, we used an extensively sampled dataset of fish and macroinvertebrate communities combined with high-resolution environmental variables (including both direct and surrogate variables) over the Loire catchment in France. Fish and macroinvertebrate communities were considered separately and together, making it possible to investigate potential differences between biological groups particularly regarding spatial concordance. First, we hypothesized that direct variables would better explain the organization of fish and macroinvertebrate communities than surrogate variables since they more accurately described the conditions experienced by organisms and their ecological preferences [28]. In addition, we hypothesized that direct variables would better explain the organization of macroinvertebrate communities than that of fish communities due to their higher sensibility to local factors, in relation to the smaller home-range size and lower dispersal capacity of macroinvertebrates [24–26]. Last, given the complexity of the relationships between aquatic communities and environmental gradients, we hypothesized the organization of the whole community (i.e. fish and macroinvertebrates combined) to reflect more accurately the biological gradient and to be better explained by environmental predictors than that of fish and macroinvertebrates taken separately [16, 18, 27].

## Materials and methods

### Study area

**Fig 1:**
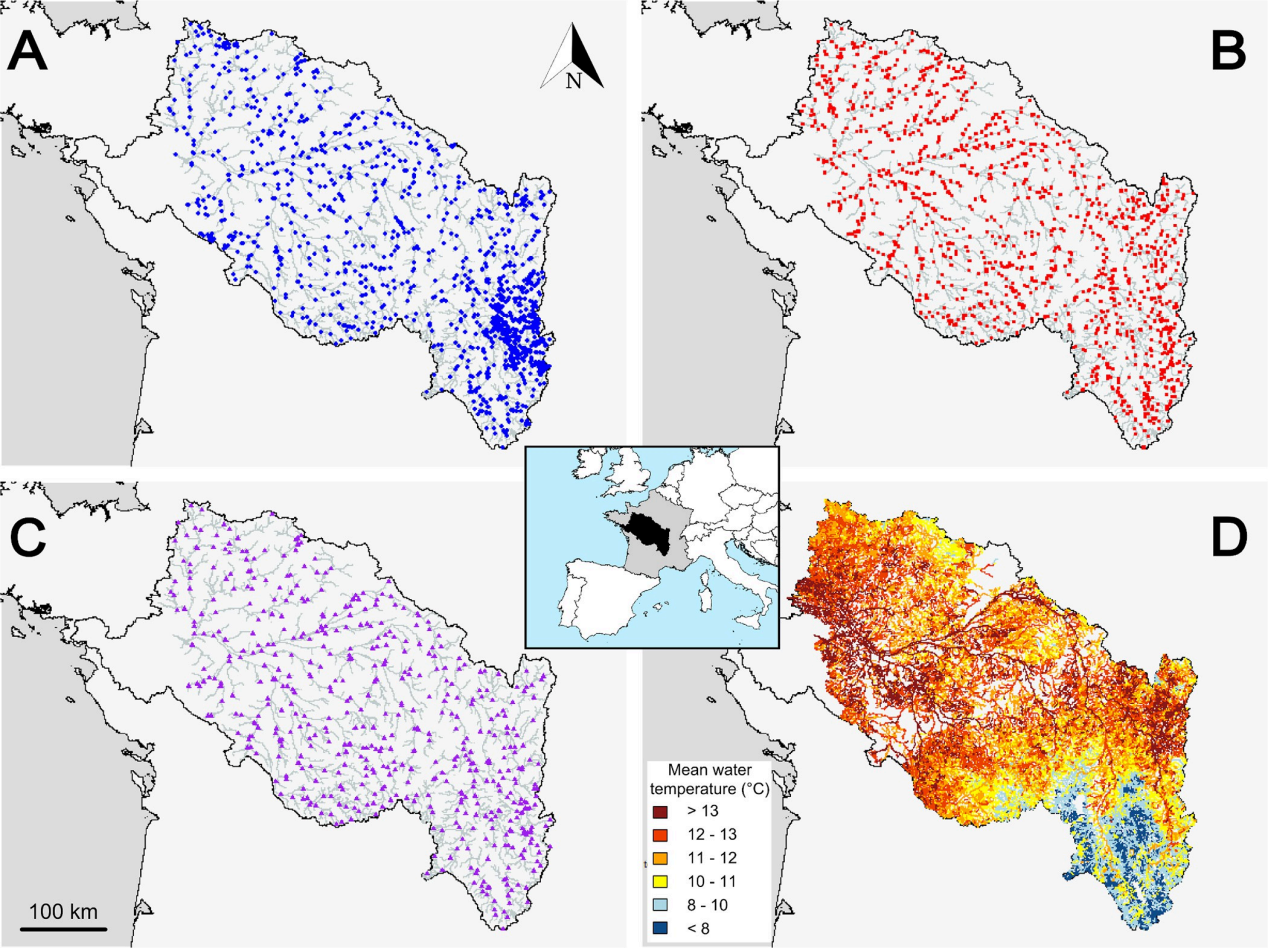
Study area and location of the sampled reaches for (A) fish, (B) macroinvertebrates and (C) both (i.e. “common” dataset) and (D) T-NET hydrographic network. In panels A, B and C, a simplified version of the T-NET hydrographic network is shown. In panel D, the 52,278 T-NET reaches are colored according to their mean annual water temperature (°C). The inserted map in the center locates the Loire catchment within Europe (source: Natural Earth).

The Loire catchment is the largest in France, with a drainage area of about 117,000 km^2^, covering 20% of the French metropolitan territory. The Loire River is 1,012 km long and runs through a variety of landscapes and climates from its source at Gerbier-de-Jonc to its estuary at Saint-Nazaire where it flows into the Atlantic Ocean. The upper part of the catchment is located within a forested mountain range (the Massif Central) while agriculture dominates in the downstream areas. The sedimentary basin is mostly occupied by cereal farming and vineyards and the western and south-eastern parts of the catchment being mainly dedicated to livestock (mostly cattle). Annual precipitation varies between 600 and 1,300 mm per year over the catchment and average air temperature ranges from 6 to 12.5°C. The Loire catchment shows a variety of hydrological regimes with eight regimes out of the 12 present in France [32, 33]. Specific discharge ranges from 3 l/s/km^2^ (in the Maine catchment) to 40 l/s/km^2^ (in the Cévennes). Seasonal variation in discharge can be high for some catchments due to very little groundwater supply [33]. The mean interannual discharge of the Loire River in its downstream part (at Montjean-sur-Loire) is 840 m^3^/s, with flood peaks 5–10 times higher (Banque Hydro; www.hydro.eaufrance.fr). The study area includes the entire Loire catchment except the part under marine influence, downstream of Montjean-sur-Loire (110 kilometers upstream from the estuary) (Fig 1).

### Environmental and biological datasets

#### Direct *vs*. surrogate environmental variables

All environmental variables were selected on the basis of knowledge of their potential influence on fish and macroinvertebrate communities (i.e. literature, expertise). A set of 23 environmental variables were considered, including descriptors of water and air temperatures, discharge, local hydraulic conditions and topography (see Table 1 for the full list of variables). They were described at the scale of the river reach (mean length ± standard deviation = 1.7 ± 1.4 kilometers), which is the spatial unit of the physical-based model Temperature-NETwork (T-NET). This model is based on a hydrographic network of the Loire catchment through 52,278 reaches and use the equilibrium temperature concept [34].

**Table 1:** Range of environmental values covered by each dataset. Average value and range (i.e. [minimum: maximum]) of the 23 environmental descriptors computed on: (1) the entire T-NET network (N = 52,278 reaches), (2) reaches with fish data (N = 1,191), (3) reaches with macroinvertebrate data (N = 1,195), and (4) reaches with fish and macroinvertebrate data (“common” dataset; N = 532). *Ta* and *Tw* refer to air and water temperature, respectively. Suffixes *m*, *sd*, *10* and *90* refer to the mean, standard deviation, lower 10% quantile and upper 90% quantile, respectively.

	Environmental variables	T-NET network	Fish	Macroinvertebrates	Common
Surrogate descriptors	Discharge m (m^3^/s)	2.9 [<0.01: 820.3]	10.2 [<0.01: 675.4]	8.5 [0.01: 675.4]	14.7 [0.01: 675.4]
	Discharge 10 (m^3^/s)	0.7 [<0.01: 212.1]	2.5 [<0.01: 171.1]	2.1 [<0.01: 171.1]	3.6 [<0.01: 171.1]
	Discharge 90 (m^3^/s)	6.2 [<0.01: 1704.9]	21.6 [<0.01: 1408.7]	18 [0.02: 1408.7]	31.2 [0.02: 1408.7]
	Discharge sd (m^3^/s)	2.7 [<0.01: 667.4]	9.2 [<0.01: 547.4]	7.9 [0.01: 547.4]	13.4 [0.01: 547.4]
	Ta m (°C)	10.7 [6.1: 12.5]	10.3 [6.2: 12.5]	10.9 [6.2: 12.5]	10.8 [6.2: 12.5]
	Ta 10 (°C)	1.9 [-2.5: 4.2]	1.4 [-2.5: 4.2]	2.0 [-2.5: 4.2]	2.0 [-2.1: 4.2]
	Ta 90 (°C)	19.4 [15.3: 21.1]	19.2 [15.3: 21.1]	19.6 [15.3: 21.1]	19.6 [15.3: 21.1]
	Ta sd (°C)	6.6 [5.5: 7.3]	6.7 [5.7: 7.3]	6.6 [5.7: 7.3]	6.7 [5.7: 7.3]
Direct descriptors	Depth m (m)	0.24 [0.02: 2.86]	0.42 [0.05: 2.61]	0.46 [0.07: 2.61]	0.55 [0.07: 2.61]
	Depth 10 (m)	0.15 [0.01: 1.79]	0.26 [0.04: 1.65]	0.28 [0.04: 1.65]	0.34 [0.05: 1.65]
	Depth 90 (m)	0.30 [0.03: 3.70]	0.53 [0.06: 3.34]	0.58 [0.08: 3.34]	0.70 [0.08: 3.34]
	Velocity m (m/s)	0.08 [<0.01: 4.14]	0.20 [<0.01: 3.78]	0.20 [0.01: 3.78]	0.28 [0.01: 3.78]
	Velocity 10 (m/s)	0.02 [<0.01: 1.23]	0.05 [<0.01: 1.08]	0.05 [<0.01: 1.08]	0.08 [<0.01: 1.08]
	Velocity 90 (m/s)	0.15 [<0.01: 7.97]	0.40 [0.01: 7.37]	0.40 [0.01: 7.37]	0.56 [0.01: 7.37]
	Width m (m)	4.6 [0.1: 204.9]	11.2 [0.5: 176.3]	11.3 [0.7: 176.3]	15.6 [0.7: 176.3]
	Width 10 (m)	4.0 [0.08: 181.2]	9.7 [0.4: 156.7]	9.8 [0.6: 156.7]	13.5 [0.6: 156.7]
	Width 90 (m)	5.0 [0.1: 219.0]	12.1 [0.5: 187.8]	12.2 [0.7: 187.8]	16.8 [0.7: 187.8]
	Tw m (°C)	11.2 [5.4: 16.2]	11.0 [5.9: 14.1]	11.5 [6.8: 14.1]	11.5 [6.8: 14.1]
	Tw 10 (°C)	6.3 [0.1: 11.5]	4.3 [0.1: 9.5]	4.8 [0.2: 8.7]	4.6 [0.5: 8.4]
	Tw 90 (°C)	17.2 [8.4: 27.6]	18.5 [10.2: 24.5]	19.1 [11.4: 24.5]	19.3 [11.4: 24.5]
	Tw sd (°C)	4.1 [0.3: 8.0]	5.3 [2.0: 7.7]	5.4 [2.7: 7.7]	5.5 [2.7: 7.7]
Others	Distance to the estuary (km)	514.5 [110.1: 1004.5]	601.0 [114.7: 999.5]	512.0 [120.2: 999.5]	511.4 [138.2: 999.5]
	Slope (‰)	0.02 [<0.01: 0.40]	0.02 [<0.01: 0.17]	0.01 [<0.01: 0.12]	0.01 [<0.01: 0.12]

Discharge data were obtained from the semi-distributed hydrological EROS model [35], which simulates daily discharge at the outlet of 368 sub-basins within the Loire catchment (sub-catchments area range from 40 to 1600 km^2^ with mean = 300 km^2^). Within a sub-basin, daily discharge is extrapolated depending on drainage area. The EROS model performed well on 44 stations at the annual scale with a mean relative bias ± standard deviation of -0.12 ± 10.62%.

Air temperature was derived from SAFRAN, which is a mesoscale atmospheric analysis system for surface variables at the hourly time step using ground data observations at the spatial resolution of a grid of 8 x 8 km [36, 37]. At annual scale the RMSE between observations and corresponding SAFRAN outputs was of 1°C [37].

Discharge statistics were translated into direct hydraulic statistics (i.e. reach width, water depth and current velocity) using the hydraulic geometry models from Morel et al. [38]. These models improve the classical hydraulic geometry equations of Leopold and Maddock [39] and relate hydraulics to discharge using power laws. To date, the models of Morel et al. [38] provide the best estimates of reach hydraulics at a given river discharge in the absence of detailed field hydraulic measurements. These models were available for a hydrographic network of the Loire catchment (Réseau Hydrographique Théorique, RHT) [40] coarser from that of the T-NET model. Therefore, we made a correspondence between RHT and T-NET river reaches, so that 49% of T-NET reaches had corresponding RHT reaches. For T-NET reaches without RHT correspondence, the hydraulic geometry coefficients were extrapolated from the nearest downstream T-NET reach with RHT correspondence.

Water temperature data were obtained from T-NET, which simulates daily water temperature for each reach of the entire river network of the Loire catchment (Fig 1D). Comparison of annual average values (i.e. the time-scale of this study) between simulations and observations at 72 stations (2010–2014) showed good performance of the T-NET model with mean bias ± standard deviation of 0.2 ± 0.82°C and mean root-mean square error (RMSE) = 0.7°C.

Based on the daily T-NET, SAFRAN and EROS outputs, we computed the interannual average of four summary statistics (i.e. annual mean, lower 10% quantile, upper 90% quantile and standard deviation) for water temperature, air temperature and discharge, respectively, in order to describe the thermal and hydrological regimes of each river reach over the period 1990–2010. Three summary statistics (i.e. annual mean, lower 10% quantile and upper 90% quantile) were calculated for hydraulic variables. Water temperature and hydraulics descriptors were considered as direct variables whereas discharge and air temperature descriptors were considered as surrogate variables. The mean slope of the river reach and the distance to the estuary, which are known to influence aquatic communities, were also considered. The mean slope of the river reach was extracted from the digital elevation model IGN BD ALTI® 25x25 m [41]. The distance to the estuary was obtained by summing the length of all T-NET reaches located downstream of the reach of interest. Metrics of discharge, hydraulic variables and slope were log-transformed prior to analyses to approach normality.

#### Fish and macroinvertebrate communities

Biological data were extracted from the French national hydrobiological monitoring database built in the context of the European Water Framework Directive [42] and the regulatory hydrobiological monitoring of five nuclear power plants (NPP) operated by Électricité de France (EDF) in the Loire catchment. Sampling operations were mostly conducted in summer and fall.

Fish data were assembled from the Naïades database [43] of the Office Français de la Biodiversité (OFB) and from the NPP database [44]. All fish samples kept for analysis were collected using standardized electrofishing protocols over the period 1994–2017 [5, 45]. The initial fish dataset consisted of abundance data for 50 species (see S1 Table for species list) collected during 5,225 fishing operations conducted in 1,393 stations. Fieldwork was conducted with adequate administrative permits for electrofishing in accordance with French laws and ethical rules.

Macroinvertebrate data were assembled from the regional databases of the Directions Régionales de l’Environnement, de l’Aménagement et du Logement (DREAL) and covered the period 1992–2015. All samples were collected following a common normalized protocol (multi-habitat sampling; norm XP T90-333; [46]). According to this protocol, eight samples were collected in different habitat types (i.e. combinations of substrates, water velocity and height) with a Surber net. Taxa identification ranged from order to genus level, but only data available at genus level were kept for consistency. The initial macroinvertebrate dataset consisted of abundance data for 230 genera collected during 4,645 sampling operations conducted at 1,215 stations (S2 Table). Abundances of fish species and macroinvertebrate genera were converted into catch per unit effort (CPUE), defined as the number of specimens per 100 m^2^ sampled (i.e. densities).

We associated the biological data to their corresponding T-NET reach by projecting sampling stations onto the T-NET hydrographic network. When stations had been sampled more than once over the study period or when two or more stations were located on the same T-NET river reach, CPUE were averaged for each taxon and species. This resulted in a total of 1,191 and 1,195 T-NET reaches sampled at least once for fish and macroinvertebrates, respectively. They were evenly distributed over the Loire catchment (Fig 1A and 1B). Finally, a third dataset was built with the 532 T-NET river reaches that included at least one sampling operation for fish and at least one for macroinvertebrates (hereafter, “common” dataset; Fig 1C). To limit the influence of the rarest species and genera, those with a frequency of occurrences of less than 0.5% were excluded from the analyses. Hence, the final dataset included the CPUE of 43 fish species and 186 macroinvertebrate genera.

### Data analysis

Non-metric multidimensional scaling (NMDS) was used to examine the organization of aquatic communities at the scale of the entire Loire catchment. This iterative ordination method is based on a ranked dissimilarity matrix between statistical samples (here T-NET reaches) and is adapted to ecological datasets including rare and low abundance taxa, especially when data from different biological groups are considered [47]. In addition, NMDS performs well in highlighting key organizational patterns at the community level, without placing too much emphasis on patterns specific to certain species or taxa. First, for each biological dataset, a dissimilarity matrix based on genera or species Wisconsin-transformed CPUE was built using Bray-Curtis dissimilarity among T-NET reaches. The best ordination was selected after 200 iterations from a random initial ordination. We assessed the quality of the NMDS using the stress value, which is a measure of the goodness-of-fit between the ranks of the observed dissimilarities and ordination distances. When stress value is lower than 0.2, the ordination is considered correct (accurate if stress values are lower than 0.1) [48].

Pairwise ordinations between biological groups were then compared using a Procrustes analysis. This method assesses the similarity of two factorial spaces and thus here the spatial concordance between biological groups. Procrustes analysis was preferred to a Mantel test because of its greater statistical power [49]. The Procrustes sum of squares *m*^2^, also called concordance value, was used to measure the similarity between two ordinations [22]. It ranges from 0 to 1 with low *m*^2^ values indicating a higher degree of similarity. Because Procrustes analyses must be conducted on ordinations performed with the same samples, NMDS were reproduced considering only the 532 T-NET reaches common to all datasets (i.e. fish, macroinvertebrate and “common”). Finally, a fourth NMDS was computed based on the 23 environmental descriptors (Table 1), for the 532 reaches common to all three biological datasets. This fourth ordination was then compared to each biological ordination using Procrustes analyses to assess the concordance between environmental gradients and the organization of each biological group.

To assess the influence of the previously described environmental variables on the organization of aquatic communities resulting from the NMDS, we perform a redundancy analysis (RDA). RDA were used as an extension of linear regression allowing to handle the multiple response variables and to overcome the collinearity inherent to the calculated metrics (i.e. mean, lower 10^th^ quantile, upper 90^th^ quantile and standard deviation) [50]. First, for each biological group (i.e. fish, macroinvertebrates and common) and each environmental variable (i.e. water temperature, air temperature, hydraulic variables and discharge), a RDA was performed with the three NMDS axes as response variables and groups of 4 metrics of the corresponding environmental variable, as explanatory variables. Second, to compare the effect of direct *vs*. surrogate variables, a variation partitioning in RDA was performed per biological group with the three NMDS axes as response variables and all metrics of direct or surrogate variables as explanatory variables (i.e. metrics of water temperature and hydraulic variables for direct variables and metrics of air temperature and discharge for surrogate variables). The respective explanatory power of each variable (coefficient of determination R^2^) for each biological group was then assessed [51]. For the hydraulic variables, for which we computed a total of nine metrics (for each of the three variables, i.e. current velocity, water depth and reach width), all combinations of four out of nine metrics were tested, to keep the same number of explanatory variables and the same degree of freedom between the RDA. To minimize the influence of spatial autocorrelation inherent to these datasets on the statistical assessments, RDA was combined with a Monte-Carlo procedure. We first selected all reaches contained in 70% of the sub-basins (i.e. among the 368 sub-basins considered in the EROS model) as calibration dataset to perform a RDA. This entire procedure was repeated 999 times. To assess both the model explanatory performance and the significance of the difference in average R^2^ values between groups of environmental variables, the overlap between confidence intervals around the R^2^ was evaluated. Confidence intervals was calculated as 2×standard deviation. The predictive performance of the model was then also assessed on the remaining 30% of sub-basins (see S1 Appendix for details).

Finally, we tested the sensitivity of the results obtained by RDA to uncertainties in environmental variables derived from physical-based models (i.e. water and air temperatures, discharge and hydraulic variables). To do so, we replicated the cross-validation procedure, except that each value of water temperature, air temperature, discharge and hydraulic variables was modified by adding a randomly sampled error scaled to the uncertainties provided for each environmental model (see S1 Appendix for details).

Data formatting and statistical analyses were performed using the R software [52]. The vegan R package [53] was used for NMDS and RDA computation and the maptools R package [54] was used for GIS data manipulation.

## Results

A total of 2,950,799 fish specimens belonging to 43 different species were sampled over the study period in the Loire catchment. This represented an average of 97 [11; 253] (mean [5^th^ and 95^th^ quantiles]) CPUEs (i.e. specimens per 100 m^2^ sampled) and 10 [1; 26] species per T-NET reach. Fish data included 18 fish families with cyprinids being the most represented (20 species). A total of 8,334,023 macroinvertebrates specimens belonging to 186 different genera were sampled over the study period. This represented an average of 3,851 [541; 10,123] CPUEs and 43 [16; 71] genera per T-NET reach. The most represented macroinvertebrate orders were: Trichoptera (55 genera), Ephemeroptera (27 genera), Coleoptera (27 genera), Gastropod (17 genera), Odonata (17 genera) and Plecoptera (14 genera). The range of environmental conditions captured by the T-NET reaches sampled was representative of the overall variety of environmental conditions found in the Loire catchment (Table 1).

**Fig 2:**
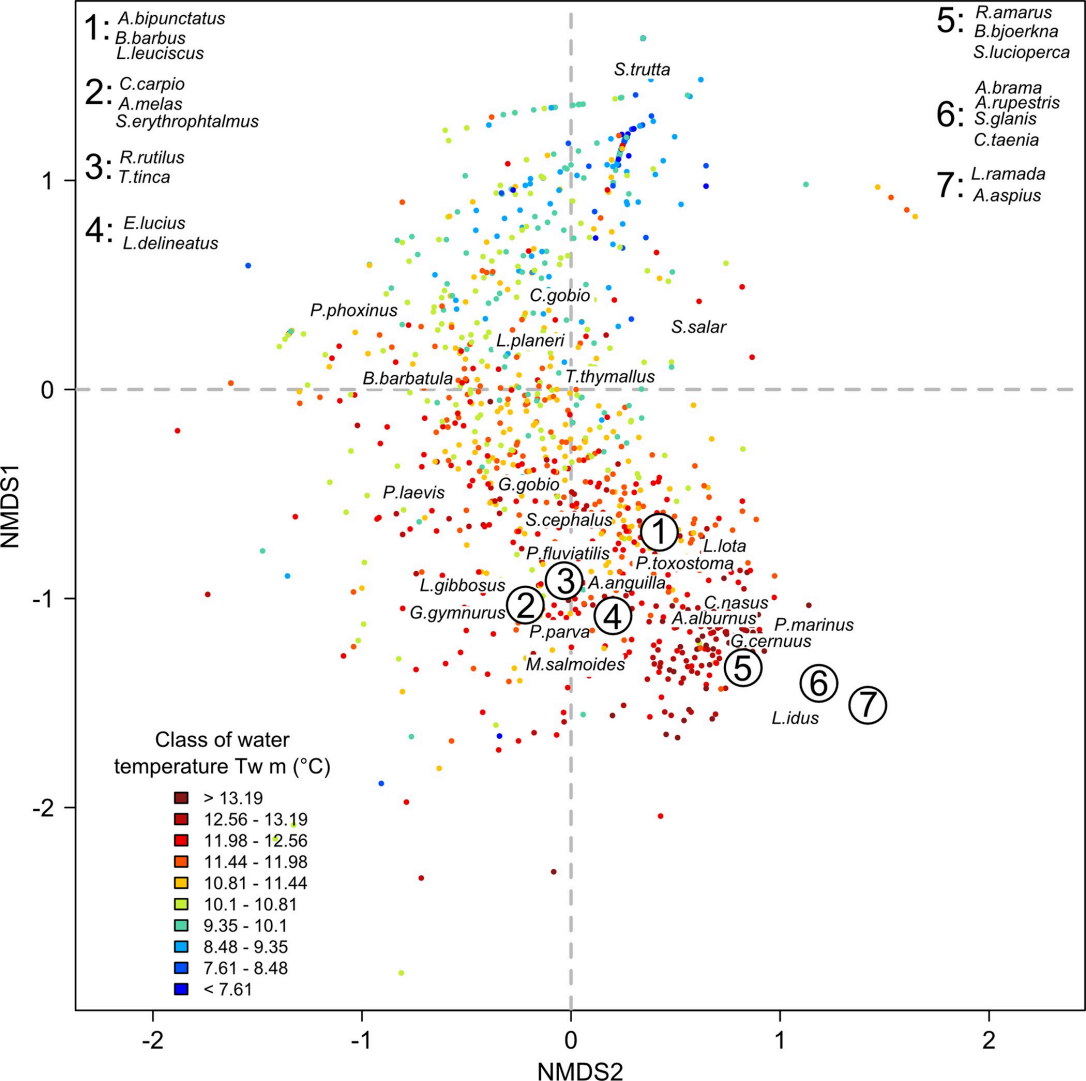
Fish ordination. Position of the T-NET reaches sampled for fish on the first factorial plane. Reaches (points; N = 1,191) are colored according to their mean water temperature (*Tw m*; in°C). Numbers indicate groups of nearby species in the factorial space, whose names have been listed in the upper corners for better readability (See S1 Table for full species names and S3 Table for the coordinates of species centroids on the three NMDS axes).

The NMDS ordination derived from the fish dataset had a stress value of 0.11, indicating a good representation (Fig 2). Fish communities with positive values along the first axis had a low species richness and were dominated by brown trout, while those with negative values had a high species richness and were composed mainly of cyprinids (S1A Fig). The NMDS ordination derived from the macroinvertebrate dataset had a stress value of 0.16, indicating a good representation (Fig 4). Macroinvertebrate communities showing positive values on the second axis were characterized by assemblages with high genera richness, while assemblages with negative values had mainly low genera richness (S1B Fig). The NMDS ordination derived from the common dataset (i.e. reaches with both fish and macroinvertebrate sampling stations) had a stress value of 0.13, indicating a good representation (Fig 5). Regarding the position of fish species centroids on the first factorial plan of the ‘common’ NMDS, the pattern was very similar to that found for the ordination based only on fish: the first axis opposed fish communities dominated by brown trout with a low taxa richness to communities composed mainly of cyprinids with a high taxa richness (S1C Fig). The pattern was not as clear for macroinvertebrates, with rich macroinvertebrate communities including abundant and common genera located near the center of the factorial space and more distinct assemblages including rarer genera located relatively far from the center.

Procrustes analyses indicated very weak concordance between each pair of biological ordinations (fish-macroinvertebrates: m^2^ = 0.99; fish-common: m^2^ = 0.99; macroinvertebrates-common: m^2^ = 0.99). The concordance with the environmental ordination was higher for the “common” ordination (m^2^ = 0.61) than for fish (m^2^ = 0.97) and macroinvertebrates (m^2^ = 0.95).

**Fig 3:**
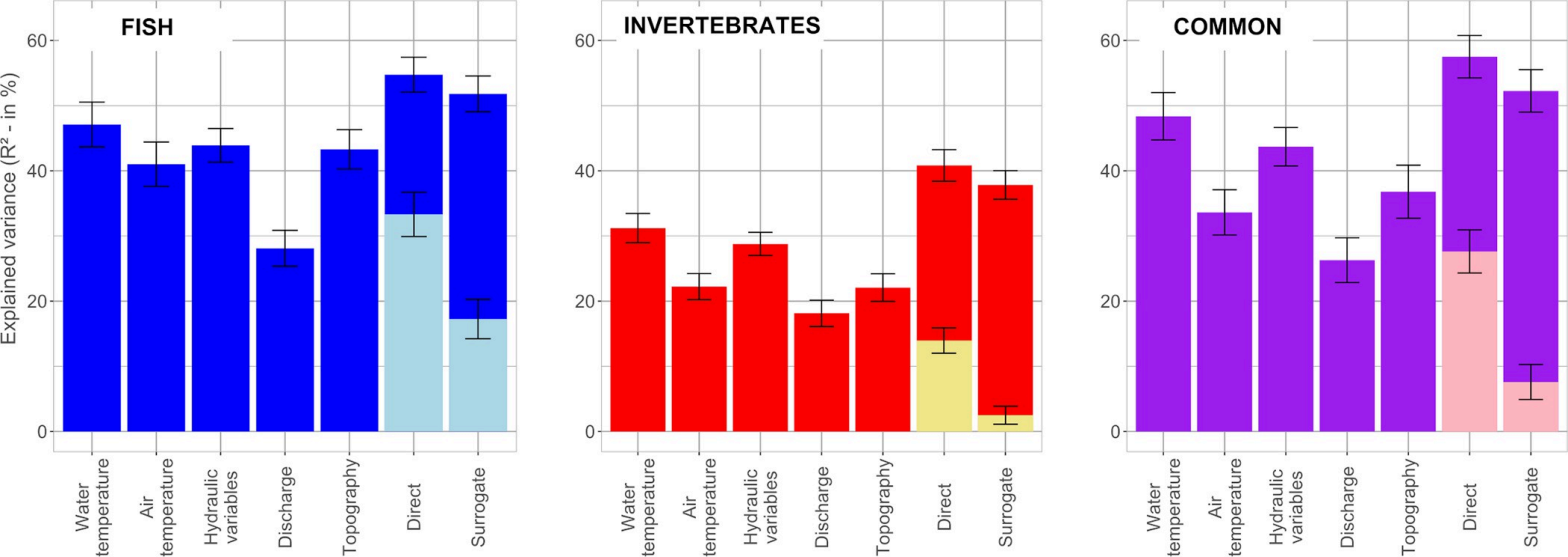
Importance of the different environmental variables in structuring the biological ordinations (i.e. fish, macroinvertebrate and common NMDS): Average coefficients of determination (R^2^; %) and associated 95% confidence interval derived from RDA (combined with variation partitioning for direct and surrogate models) and obtained through 999 random sub-samples of reaches contained in 70% of sub-basins. Shared fractions of explained variance are shown in lighter color within the bars corresponding to direct and surrogate models.

Fish ordination was mainly organized along the upstream-downstream gradient as illustrated by mean water temperature (Fig 2). Based on RDA (Fig 3), the most structuring variables of the fish ordination were, by order of importance, water temperature (average explanatory R^2^ across the 999 iterations = 47.1%), hydraulic variables (R^2^ = 43.9), slope and distance to the estuary (R^2^ = 43.3), air temperature (R^2^ = 41.0) and discharge (R^2^ = 28.1). When considered together, direct variables explained the fish ordination with an R^2^ = 54.7 and the surrogate variables with an R^2^ = 51.8.

**Fig 4:**
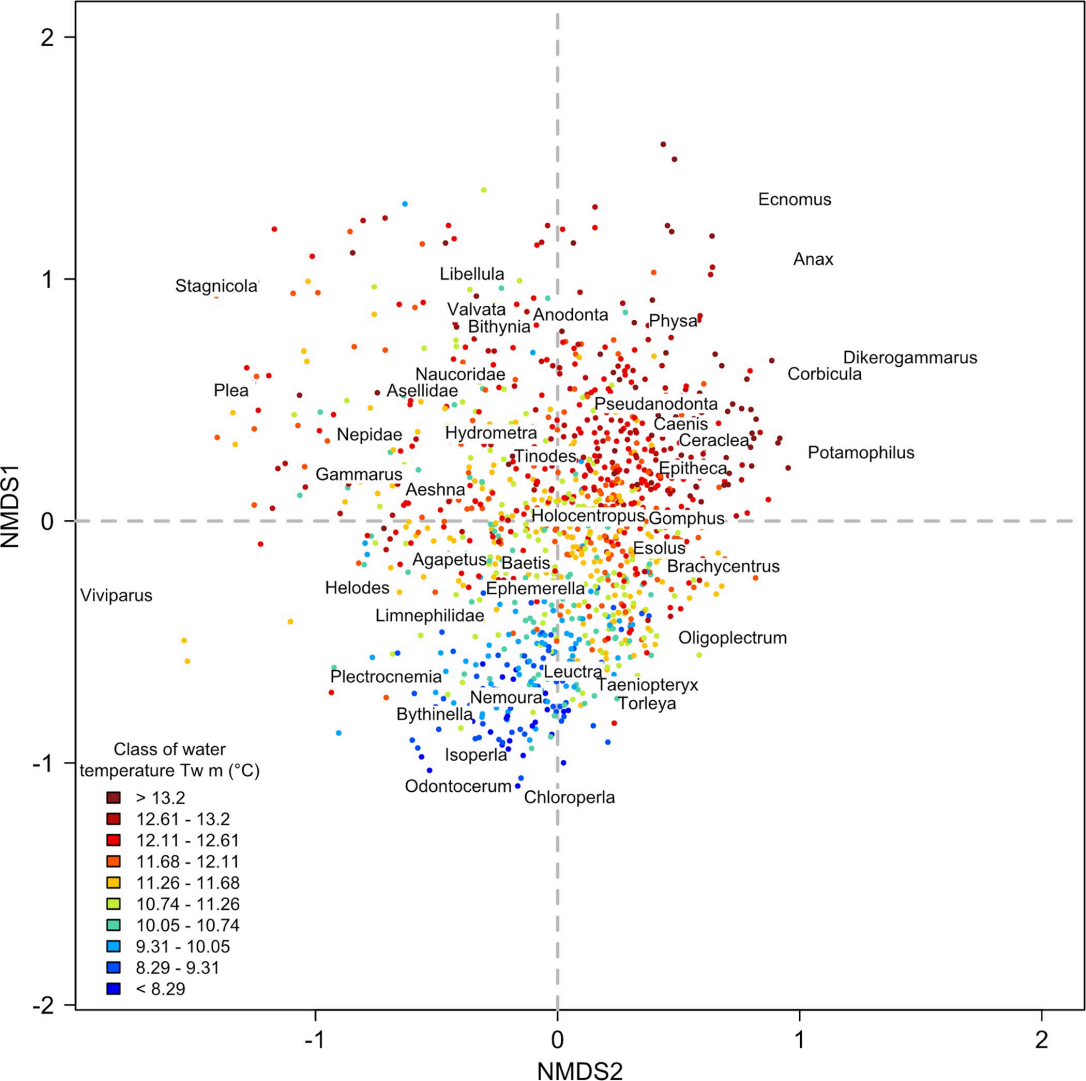
Macroinvertebrate ordination. Position of the T-NET reaches sampled for macroinvertebrates on the first factorial plane. Reaches (points; N = 1,195) are colored according to their mean water temperature (*Tw m*; in°C). For better readability, only centroids of the most represented macroinvertebrate genera are displayed (see S2 Table for correspondence between code and taxon name and S4 Table for the coordinates of all the genera centroids on the three NMDS axes).

Considering macroinvertebrate ordination, the structuration by the main environmental gradients was visible (e.g. thermal gradient, Fig 4) but appeared less clearly than for fish. Based on RDA (Fig 3), the most structuring variables of the macroinvertebrate ordination were, by order of importance, which is the same than for fish, water temperature (R^2^ = 31.2), hydraulic variables (R^2^ = 28.8), air temperature (R^2^ = 22.2), slope and distance to the estuary (R^2^ = 22.1) and discharge (R^2^ = 18.1). The direct variables taken together explained the macroinvertebrate ordination with an R^2^ = 40.8 and the surrogate variable with an R^2^ = 37.8.

**Fig 5:**
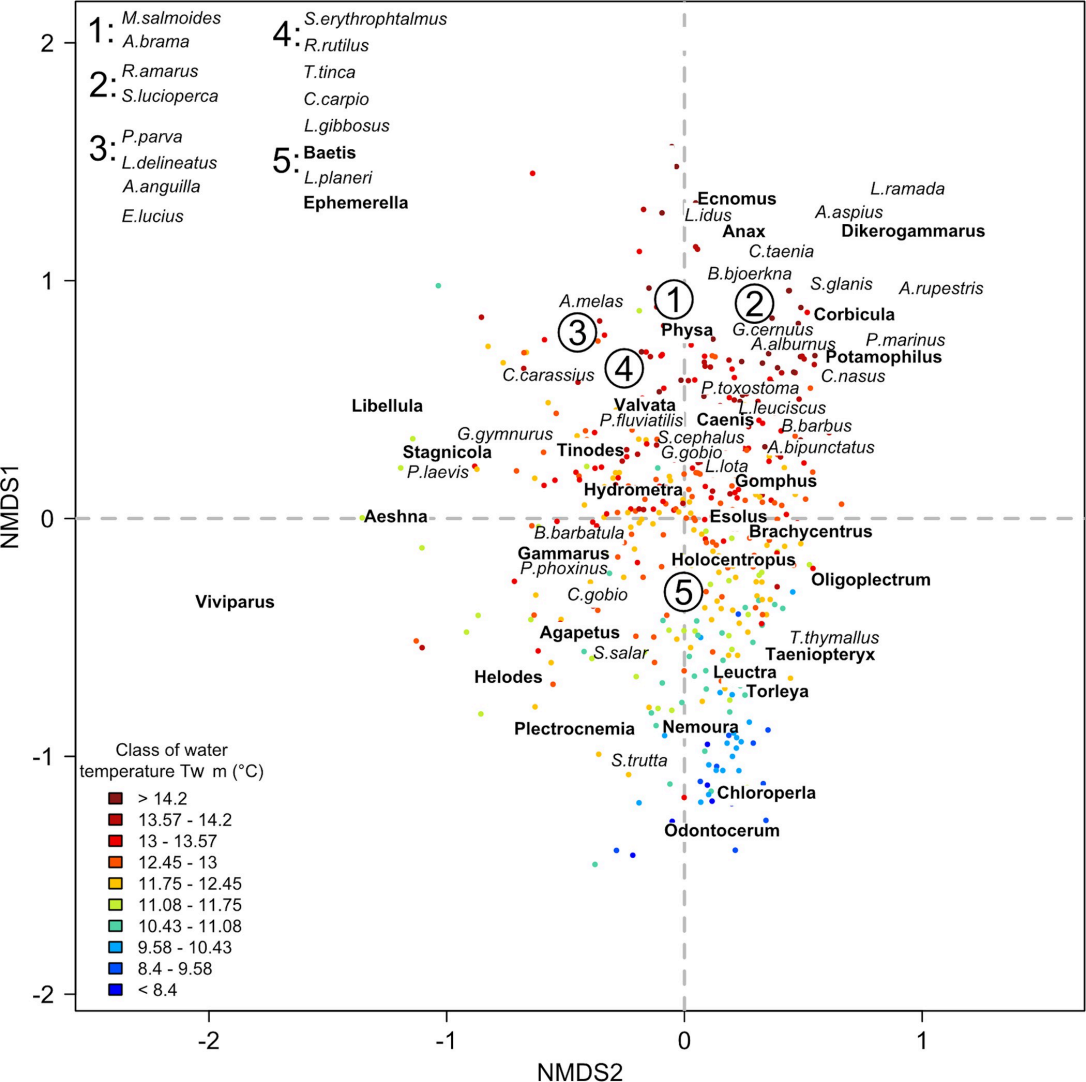
Common ordination. Position of the T-NET reaches sampled for both fish and macroinvertebrates on the first factorial plane. Reaches (points; N = 532) were colored according to their mean water temperature (*Tw m*; in°C). Numbers indicate groups of nearby fish species (in italics) or macroinvertebrate genera (in bold) in the factorial space, whose names have been listed in the upper left corner for better readability. See S1 and S2 Tables for full species and genera names. See S5 Table for the coordinates of all species and genera centroids on the three NMDS axes.

The ordination produced using the common dataset (i.e. with fish and macroinvertebrate considered together, Fig 5) showed similarities with fish and macroinvertebrate ordinations, both in terms of placement of reaches relative to each other and placement of taxon centroids. Based on RDA (Fig 3), the most structuring variables of the common ordination, by order of importance, were water temperature (R^2^ = 48.4), hydraulic variables (R^2^ = 43.7), slope and distance to the estuary (R^2^ = 36.8), air temperature (R^2^ = 33.6) and discharge (R^2^ = 26.3). The direct variables taken together explained the common ordination with an R^2^ = 57.5 and the surrogates variables with an R^2^ = 52.3.

Regarding the explanatory power of RDA, the difference in R^2^ between each pair of direct and surrogate variables (i.e. water temperature vs. air temperature, hydraulic variables vs. discharge, and direct vs. surrogate combined) were all statistically significant, as shown by the non-overlapping confidence intervals (only with means for the direct and surrogate models), with the direct variables explaining all three biological ordinations better than the surrogate variables. Regarding the predictive power of RDA, most R^2^ were also higher for the direct than for the surrogate variables, although the differences were mostly non-significant due to wider confidence intervals (S1 Appendix). Finally, these results were found to be robust to uncertainties in the environmental variables derived from physical-based models (i.e. water and air temperatures, discharge and hydraulic variables), according to the sensitivity analysis (S1 Appendix).

## Discussion

### Main environmental determinants of the organization of aquatic communities in the Loire catchment

The NMDS performed on the three biological datasets (i.e. fish, macroinvertebrates and common) highlighted the role of the longitudinal gradient in structuring aquatic communities at the extent of a large catchment. The NMDS based on fish densities closely transcribed this longitudinal gradient, the relationships with the environmental variables showing the opposition between thermal and hydraulic-hydrological variables (increasing from upstream to downstream) and topographic variables (decreasing). Fish communities varied along this axis, from salmonid-dominated communities in the upstream reaches to cyprinid-dominated communities in the downstream areas, with a concomitant increase in species richness and total density. The resulting organization of fish communities is consistent with that generally described in the literature [15, 20]. The relationship between the upstream-downstream environmental gradient and macroinvertebrate densities was less clear but still appeared on the second NMDS axis. All R^2^ between the macroinvertebrate ordination and the environmental variables were slightly lower than those found for fish, which indicates that the higher variability of the macroinvertebrate community was slightly less explained by our set of environmental variables. This suggests that macroinvertebrate communities are not as strongly structured by direct variables and the longitudinal gradient at this spatial extent as fish communities are. Key differences in the ecology of fish and macroinvertebrates may explain this result. For example, the greater ability of fish have access to more diverse habitats and can therefore occupy habitats more closely aligned with their ecological requirements [26, 55] Overall, for the three biological NMDS, we did not observe clustered groups of reaches or any other patchy patterns, although the sampling stations covered very distinctive geographical entities (e.g. Massif Central, Loire valley). This supports the idea that succession between communities is gradual and continuous, with transitions where species with different ecological preferences co-exist, rather than being bounded (as formulated in the River Continuum Concept) [18].

The community organization of the aquatic groups studied was essentially explained by the same set of environmental variables. The most influential determinants were water temperature (especially mean and quantile 90), hydraulic variables (i.e. water depth, reach width and current velocity) and slope associated to the distance to estuary. This finding is consistent with the literature on the topic. First, water temperature, especially warm temperature, limits the spatial distribution of aquatic species according to their thermal tolerance and oxygen requirements [28, 56, 57]. Nonetheless, while being frequently recognized in the literature, few studies have shown the key role of water temperature in structuring aquatic communities at large scale using water rather than air temperature data. Second, hydraulic conditions represent an important local environmental filter that constrains species depending on their hydraulic habitat preferences, also influencing other habitat characteristics (e.g. substrate size, type of vegetation) [58, 59].

### Direct vs. surrogate environmental drivers of the organization of aquatic communities

We found that direct environmental variables (i.e. water temperature and hydraulic descriptors) explained significantly better the organization of aquatic communities at the scale of the Loire catchment than surrogate ones (i.e. air temperature and discharge). This corroborates our first hypothesis assuming that direct variables better reflect the actual conditions experienced by aquatic organisms than surrogate ones, therefore better explaining their community organization. We found significant differences between water and air temperature in their ability to explain the organization of fish and macroinvertebrate communities. Studies generally agreed that water temperature is highly correlated with air temperature, especially when the spatial resolution of the study is large [28]. As water temperature measurements are rarely available at large extent, air temperature data are therefore often used as a proxy in large extent ecological studies (e.g. [5, 60, 61]). However, air temperature does not capture all the variability of water temperature. This is highlighted by the ranges of values taken by the two kind of metrics at the scale of the whole T-NET network (see Table 1), with for instance mean air temperature ranging from 6.1 to 12.5°C whereas mean water temperature ranges from 5.4 to 16.2°C (the differences being even higher for the values of lower 10% quantile and upper 90% quantile). Consequently, air temperature does not substitute well for water temperature under certain conditions, such as in the most upstream reaches or near groundwater inputs [28, 34]. For all three biological datasets, the relationships between the NMDS ordinations and the discharge were weaker than with the corresponding hydraulic variables. This supports the idea that the resolution at which discharge metrics have been computed was not the most appropriate to correctly transcribe the relationships between organisms and discharge, unlike hydraulic metrics, which describe habitats at a finer grain. Indeed, hydraulic metrics provide additional dimensions relatively to discharge, because they better reflect local geomorphological variations that influence habitats [38, 62, 63]. Finally, when the direct environmental variables were considered together, they still better explained the organization of fish and macroinvertebrate communities than the full set of surrogate environmental variables. Moreover, the differences between direct and surrogate variables were even greater for the common dataset. These two results reinforce the hypothesis that the combination of several biological groups associated to the use of direct environmental variables is most suitable for accurately assessing the environmental-organism relationships in lotic hydrosystems. As far as possible, we recommend using several biological groups and direct variables to study spatial patterns of aquatic communities. Nonetheless, surrogate variables remain valuable when direct variables are not available, especially at large scale where the differences in explaining the organization of aquatic communities can be slight as found in the present study. In particular, direct variables are likely to perform better at finer spatial extents and may be more interesting for environmental management as they are more directly linked to actual levers of action. These differences were further reduced with respect to the predictive power of both variables. Therefore, the choice of using direct or surrogate environmental variables may also depend on whether the objective is to explain and understand the organization of aquatic communities and its main drivers, or rather to predict spatial patterns under different scenarios (e.g. climate change).

The environmental variables studied here only partially explained the organization of fish and macroinvertebrate communities. The spatial resolution of the study was the T-NET reach (~1.7 km on average; N = 52,278) and we believe that such accuracy, density and coverage at the extent of a large hydrographic network (cumulative length of the river system = 88,058 km) made it highly suitable for precisely investigating the organization of aquatic communities in the Loire basin. Setting the study at a broad spatial extent allows capturing organism responses to major environmental gradients arising from climate, geology or topography [64]. Nevertheless, some processes occurring at a markedly different spatial scale may be blurred at such large spatial extent [65, 66]. For instance, large spatial extent may include distinct biogeographic units, which are known to be driven by other factors such as historical events (*e*.*g*. major glaciations) and dispersal capacity of organisms [67–69]. While the catchment scale is now widely recognized as appropriate for examining the organization of fish communities and for management and conservation measures (e.g. Huet [12]; Miranda et al. [70]; Oberdorff et al. [69]), the appropriate extent at which macroinvertebrate communities should be studied remains under debate [71]. For example, Hawkins and Norris [72] and Johnson et al. [73] agreed that the organization of macroinvertebrate communities is better explained by local habitat factors (e.g. substrate, aquatic vegetation) than coarser ones (e.g. land-use in the upstream drainage area, position of the stream in the hydrographic network) whereas Townsend et al. [74] found the opposite. Overall, it seems that the organization of aquatic communities is ruled by the interplay of different spatial scales, with broad-scale processes influencing those occurring at finer scales [17, 58, 73, 75, 76]. One way to capture these scale interactions would be to build hierarchical models able to account for several scales together (e.g. Villeneuve et al. [17]). Here, the NMDS based on macroinvertebrate densities suggested the role of processes occurring at two levels: catchment scale, governed mainly by thermal and hydrologic regimes, and reach scale at which hydraulic variables show important variations. However, explanatory variables that may be in interaction with variables already considered may have been overlooked, and their inclusion could improve our understanding of the organization of aquatic communities. Furthermore, limitations in the modelling of environmental variables may also influence their contribution in explaining the organization of aquatic communities. The sensitivity analysis performed (S1 Appendix) showed that the results were robust to the uncertainties arising from the physical-based environmental models. Nevertheless, we acknowledge that the T-NET and EROS models, from which the water temperature and discharge data used in this study are derived, do not account for all human-induced alterations of streams, notably physical barriers (e.g. dams, weirs) that are known to alter the hydrological and thermal regimes of rivers [77, 78]. Although the Loire basin is acknowledged as one of the last wild river in Europe because of a limited impact of human activities on its hydrological regime, further developments to integrate anthropogenic uses would be interesting [33].

### Concordance between the organization of fish and macroinvertebrate communities and the value of a cross-taxa approach

The concordance between the three NMDS ordinations (*i*.*e*. fish, macroinvertebrates and “common”) was very weak. This result highlighted that fish and macroinvertebrate communities do not seem to share very similar spatial patterns at the extent of the Loire catchment. Thus, while being driven by the same set of environmental variables, both communities seem to respond differently to environmental gradients. This reinforces the assumption that macroinvertebrate and fish communities are sensitive to environmental gradients but at a different spatial extent. The weak concordance between their community organization confirms the relevance of studying fish and macroinvertebrates together to identify generalizable distribution patterns of aquatic communities instead of considering only one or the other. Most studies that used a similar approach (i.e. multivariate ordination and concordance assessment) have also found weak concordances between biological groups at spatial extents similar to the Loire catchment (e.g. fish and macroinvertebrates in Kilgour and Barton [79]; many biological groups in the review from Heino [24]; diatoms, macroinvertebrates, macrophytes and fish in Johnson and Hering [80]). In addition, some studies have compared the concordance between taxonomic groups at different spatial scales. Dolph et al. [23] and Paavola et al. [22] found greater concordance in the organization of fish and macroinvertebrate communities at broader spatial extent(i.e. similar to the Loire catchment) whereas Bae et al. [81] found the opposite. The first two studies showed that fish and macroinvertebrate communities were concordant at broad geographical scale, over study areas of similar size to that of the Loire catchment (i.e. 101 stations within two ecoregions in Finland in Paavola et al. [22] and 670 stations in Minnesota, USA in Dolph et al. [23]). However, there are noteworthy differences between these studies and the present one. Paavola et al. [22] focused on boreal aquatic communities, which live under much colder conditions and have lower richness and abundance. Dolph et al. [23] based their results on concordance between taxonomic groups using species-poor North American communities with a total of 5 fish species and 15 macroinvertebrates taxa. Moreover, in both studies, the broad spatial extent is constituted by grouping small sampling areas from different distant sub-basins, thus not considering a single large catchment that would include the full range of environmental conditions. Furthermore, the weak concordance found between the ordination based on the “common” dataset (i.e. combining fish and macroinvertebrate sampling) and the other ordinations indicated that neither fish nor macroinvertebrates were more representative of the organization of the combined aquatic communities. The influence of environmental drivers differed: macroinvertebrates showed weaker correlations with most environmental variables than fish, despite our second hypothesis. The concordance between the environmental ordination and the “common” ordination was higher than with fish and macroinvertebrate ordinations, which is certainly due to a better embedding of the environmental gradient through the biological gradient captured by the NMDS combining fish and macroinvertebrate data (i.e. richer and more complex communities). This observation was also confirmed by the slightly higher R^2^ found between most explaining environmental variables and the “common” ordination. This supports our third hypothesis that combining different biological groups improves the fit with the main environmental gradients, even though it increases the biological variability and complexity.

Some hypotheses may explain the differences between biological groups found here. First, the macroinvertebrate dataset included 186 genera against 43 species for fish. Consequently, communities were more diverse for macroinvertebrates than for fish (on average 43 genera per reach for macroinvertebrates *vs*. 10 species per reach for fish). The observed CPUE were also more variable for macroinvertebrates (CPUE mean = 3,851 ± 3,121 specimens per 100 m^2^ per T-NET reach) than for fish (CPUE mean = 97 ± 140 specimens per 100 m^2^ per T-NET reach). This could lead to a higher variability to be explained by the same set of environmental variables for macroinvertebrates than for fish. Furthermore, the extent of the spatial distribution of macroinvertebrate genera was on overall more variable than for fish species, with ubiquitous genera occurring along the whole longitudinal gradient (e.g. the genus *Baetis* was present in more than 95% of the reaches sampled; S2 Table) and other genera showing very particular ecological preferences and restricted occurrences within the Loire catchment (e.g. the genus *Leptophlebia* was present in less than 2% of the reaches sampled, mainly in upstream reaches; S2 Table). In contrast, fish ordination has highlighted typical ‘indicator’ species like *S*.*trutta* or *T*.*thymallus* whose ecological requirements are well known and which are found in specific parts of the Loire catchment. Considering the high number of genera in the macroinvertebrate dataset and the large extent of the area studied, it seems plausible that the Loire catchment includes distinct sub-basins, each having distinct macroinvertebrate biogeographic patterns. The organization of macroinvertebrate communities would thus be less driven by the upstream-downstream environmental gradient. In contrast, the longitudinal organization of fish assemblages seems to apply to the entire catchment. Second, the identification of macroinvertebrates was limited to the genus level, which may group species with different ecological preferences [72, 82]. Previous studies had shown that family-level data can be sufficient to examine the main spatial patterns in macroinvertebrate communities, although more precise patterns can only be detected with a finer level of taxonomic identification [83]. In addition, the sensitivity of non-metric ordination methods to the identification level of macroinvertebrates is known to be reduced under the family level [55, 79]. According to these studies, the use of the genus level seems to be a good compromise between practical limitations (i.e. field surveys, time allocated to taxa identification) and appropriate identification level to examine ecological patterns. Third, fish typically use larger areas of stream to complete their life cycle than macroinvertebrates: above the kilometer for most fish species *vs*. a few meters for most macroinvertebrate genera [26, 79]. Both groups can therefore be sensitive to the same environmental variables but not at the same spatial grain [84]. Macroinvertebrates are more sensitive to local determinants (*sensu* habitat variables) and show stronger responses to local perturbations than fish, especially due to their lower dispersal and avoidance abilities in their larval form [58, 85, 86]. This implies that the identification of patterns in the organization of macroinvertebrate communities at large extent might be blurred by local processes and disturbances acting at fine grain. Fish are probably less affected by these fine-grained factors as they are able to move to suitable habitats when habitat alteration is local and temporary. Stochastic processes of colonization dynamic (e.g. organisms’ drifts, flying macroinvertebrates) may also play a more important role for macroinvertebrates than for fish [26].

## Conclusion and perspectives

Using high-resolution environmental datasets in a large catchment, we demonstrated the ability of direct environmental descriptors to better explain the organization of fish and macroinvertebrate communities. We also addressed the concordance between the organization of fish and macroinvertebrate communities and identified common environmental determinants. We found that fish and macroinvertebrates exhibited weak concordance in their community organization due to their different responses to ecological gradients, which precludes using one biological group as a proxy for the other. Future research should continue to examine together data from different taxonomic groups, paying particular attention to the role of spatial scale (i.e. extent and resolution), and pursue the efforts to make accurate, fine-grained environmental variables available through monitoring and modelling over large spatial extents. Including anthropogenic influences in environmental models (e.g. T-NET, EROS) is an interesting avenue to grasp more environmental variability and to gain insights into the spatial patterns of aquatic communities, in line with recent meta-community concepts. Thus, improving our understanding of how communities are organized in a changing environment (i.e. climate change) is of primary importance for field managers and biodiversity stakeholders.

## Supporting information

S1 Fig Taxonomic richness of the sampled T-NET reaches plotted on the first factorial plan of the fish, macroinvertebrates and common ordinations (A, B and C, respectively). Reaches are colored according to their taxonomic richness from low (blue) to high (red) number of species or genera.
(PDF)

S1 Table List of 50 fish species present in the initial dataset. For each species, the number of occurrences (with the frequency of occurrence under brackets) over the 1191 T-NET reaches sampled is provided, as well as the average CPUE per reach (in number of specimens per 100 m^2^ sampled). The species marked with an asterisk correspond to the rarest species (frequency of occurrence < 0.5%) that were excluded prior to analyses.
(PDF)

S2 Table List of 230 macroinvertebrates genera present in the initial dataset. For each taxa, the number of occurrences (with the frequency of occurrence under brackets) over the 1195 T-NET reaches sampled is provided, as well as the average CPUE per reach (in number of specimens per 100 m^2^ sampled). The taxa marked with an asterisk correspond to the rarest taxa (frequency of occurrence < 0.5%) that were excluded prior to analyses.
(PDF)

S3 Table Position of the centroids of the 43 fish species on the three factorial axes of the NMDS performed on the fish dataset. (PDF)

S4 Table Position of the centroids of the 186 macroinvertebrates taxa on the three factorial axes of the NMDS performed on the macroinvertebrate dataset. (PDF)

S5 Table Position of the centroids of the 43 fish species (bold) and 170 macroinvertebrates taxa (non-bold) on the three factorial axes of the NMDS performed on the common dataset. (PDF)

S1 Appendix Monte Carlo cross-validation procedure and sensitivity analysis of RDA to uncertainties in environmental variables derived from physical-based models. (PDF)
