# Supplementary material for: Direct habitat descriptors improve the understanding of the organization of fish and macroinvertebrate communities across a large catchment
Source: PLoS One. 2022 Sep 22;17(9):e0274167. doi: 10.1371/journal.pone.0274167 (PMC9498974; doi:10.1371/journal.pone.0274167)
Supplement: S1 Fig — Taxonomic richness of the sampled T-NET reaches plotted on the first factorial plan of the fish, macroinvertebrates and common ordinations (A, B and C, respectively). Reaches are colored according to their taxonomic richness from low (blue) to high (red) number of species or genera. (PDF) [file pone.0274167.s001.pdf]

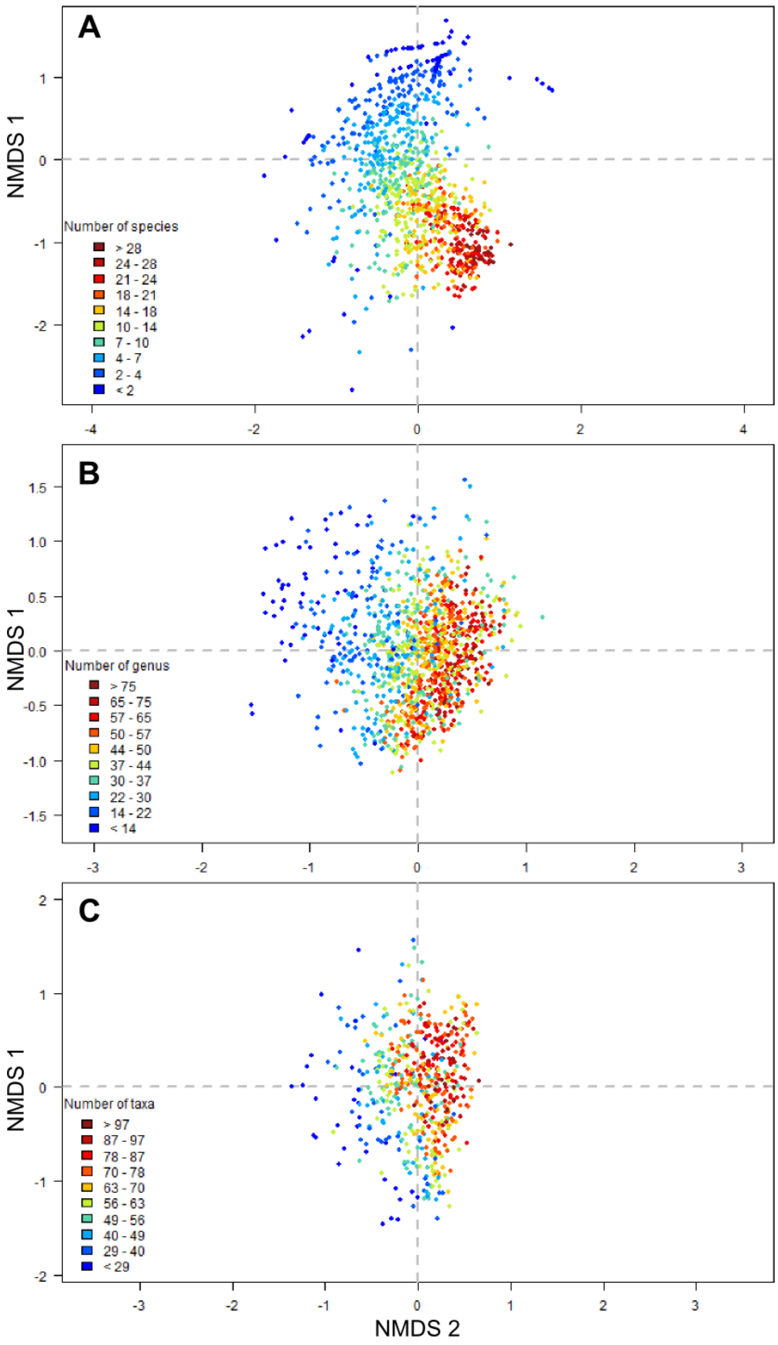

S1 Figure. Taxonomic richness of the sampled T-NET reaches plotted on the first factorial plan of the fish, macroinvertebrates and common ordinations (A, B and C, respectively). Reaches are colored according to their taxonomic richness from low (blue) to high (red) number of species or genera.
