# Supplementary material for: Direct habitat descriptors improve the understanding of the organization of fish and macroinvertebrate communities across a large catchment
Source: PLoS One. 2022 Sep 22;17(9):e0274167. doi: 10.1371/journal.pone.0274167 (PMC9498974; doi:10.1371/journal.pone.0274167)
Supplement: S1 Table — For each species, the number of occurrences (with the frequency of occurrence under brackets) over the 1191 T-NET reaches sampled is provided, as well as the average CPUE per reach (in number of specimens per 100 m2 sampled). The species marked with an asterisk correspond to the rarest species (frequency of occurrence < 0.5%) that were excluded prior to analyses. (PDF) [file pone.0274167.s002.pdf]

S1 Table. List of 50 fish species present in the initial dataset. For each species, the number of occurrences (with the frequency of occurrence under brackets) over the 1191 T-NET reaches sampled is provided, as well as the average CPUE per reach (in number of specimens per 100 m² sampled). The species marked with an asterisk correspond to the rarest species (frequency of occurrence < 0.5%) that were excluded prior to analyses.

Scientific name	Family	Occurrences (frequency)	Average CPUE per reach (ind/100 m²)
Abramis brama	Cyprinidae	228 (19.1%)	0.12
Alburnoides bipunctatus	Cyprinidae	363 (30.5%)	3.54
Alburnus alburnus	Cyprinidae	348 (29.2%)	2.37
*Alosa alosa**	Clupeidae	1 (0.1%)	< 0.01
*Alosa fallax fallax**	Clupeidae	1 (0.1%)	< 0.01
Ambloplites rupestris	Centrarchidae	11 (0.9%)	0.01
Ameiurus melas	Ictaluridae	269 (22.6%)	0.64
*Ameiurus nebulosus**	Ictaluridae	3 (0.3%)	< 0.01
Anguilla anguilla	Anguillidae	358 (30.1%)	0.23
Aspius aspius	Cyprinidae	21 (1.8%)	< 0.01
Barbatula barbatula	Nemacheilidae	818 (68.7%)	8.72
Barbus barbus	Cyprinidae	349 (29.3%)	1.29
Blicca bjoerkna	Cyprinidae	192 (16.1%)	0.3
Chondrostoma nasus	Cyprinidae	184 (15.4%)	0.22
Cobitis taenia	Cobitidae	25 (2.1%)	0.02
Cottus gobio	Cottidae	579 (48.6%)	9.04
Cyprinus carpio	Cyprinidae	277 (23.3%)	0.12
Esox lucius	Esocidae	304 (25.5%)	0.07
Gasterosteus gymnurus	Gasterosteidae	50 (4.2%)	0.2
Gobio gobio	Cyprinidae	744 (62.5%)	9.62
Gymnocephalus cernuus	Percidae	177 (14.9%)	0.05
*Hypophthalmichthys molitrix**	Cyprinidae	4 (0.3%)	< 0.01
*Lampetra fluviatilis**	Petromyzontidae	4 (0.3%)	< 0.01
Lampetra planeri	Petromyzontidae	374 (31.4%)	1.34
Lepomis gibbosus	Centrarchidae	479 (40.2%)	1.47
Leucaspius delineatus	Cyprinidae	105 (8.8%)	0.02
Leuciscus idus	Cyprinidae	10 (0.8%)	< 0.01
Leuciscus leuciscus	Cyprinidae	311 (26.1%)	0.3
Liza ramada	Mugilidae	16 (1.3%)	< 0.01
Lota lota	Gadidae	51 (4.3%)	0.01
Micropterus salmoides	Centrarchidae	45 (3.8%)	0.01
Carrassius sp.	Cyprinidae	234 (19.6%)	0.13
Parachondrostoma toxostoma	Cyprinidae	19 (1.6%)	0.01
Perca fluviatilis	Percidae	534 (44.8%)	1.03
Petromyzon marinus	Petromyzontidae	56 (4.7%)	0.01
Phoxinus phoxinus	Cyprinidae	801 (67.3%)	27.52
*Platichthys flesus**	Pleuronectidae	1 (0.1%)	< 0.01
Pseudorasbora parva	Cyprinidae	221 (18.6%)	0.56

Pungitius laevis	Gasterosteidae	177 (14.9%)	1.88
Rhodeus amarus	Cyprinidae	285 (23.9%)	1.72
Rutilus rutilus	Cyprinidae	602 (50.5%)	5.22
Salmo salar	Salmonidae	59 (5.0%)	0.29
Salmo trutta	Salmonidae	788 (66.2%)	12.35
Sander luciopercia	Percidae	154 (12.9%)	0.02
Scardinius erythrophthalmus	Cyprinidae	345 (29.0%)	0.43
Silurus glanis	Siluridae	126 (10.6%)	0.05
Squalius cephalus	Cyprinidae	679 (57.0%)	5.42
*Telestes souffia**	Cyprinidae	2 (0.2%)	< 0.01
Thymallus thymallus	Salmonidae	53 (4.5%)	0.02
Tinca tinca	Cyprinidae	306 (25.7%)	0.11
