# Supplementary material for: Direct habitat descriptors improve the understanding of the organization of fish and macroinvertebrate communities across a large catchment
Source: PLoS One. 2022 Sep 22;17(9):e0274167. doi: 10.1371/journal.pone.0274167 (PMC9498974; doi:10.1371/journal.pone.0274167)
Supplement: S2 Table — For each taxa, the number of occurrences (with the frequency of occurrence under brackets) over the 1195 T-NET reaches sampled is provided, as well as the average CPUE per reach (in number of specimens per 100 m2 sampled). The taxa marked with an asterisk correspond to the rarest taxa (frequency of occurrence < 0.5%) that were excluded prior to analyses. (PDF) [file pone.0274167.s003.pdf]

S2 Table. List of 230 macroinvertebrates genera present in the initial dataset. For each taxa, the number of occurrences (with the frequency of occurrence under brackets) over the 1195 T-NET reaches sampled is provided, as well as the average CPUE per reach (in number of specimens per 100 m² sampled). The taxa marked with an asterisk correspond to the rarest taxa (frequency of occurrence < 0.5%) that were excluded prior to analyses.

Genus	Order	Family	Occurrences (frequency)	Average CPUE per reach (ind/100 m²)
Acentrella	Ephemeroptera	Baetidae	30 (2.5%)	0.24
Acroloxus	Gastropoda	Acroloxidae	158 (13.2%)	0.52
Adicella	Trichoptera	Leptoceridae	286 (23.9%)	1.27
Aeshna	Odonata	Aeshnidae	7 (0.6%)	< 0.01
Agapetus	Trichoptera	Glossosomatidae	239 (20.0%)	6.8
Agraylea	Trichoptera	Hydroptilidae	37 (3.1%)	0.16
Allotrichia	Trichoptera	Hydroptilidae	7 (0.6%)	0.02
*Ameletus**	Ephemeroptera	Ameletidae	2 (0.2%)	< 0.01
*Ametropus**	Ephemeroptera	Ametropodidae	1 (0.1%)	< 0.01
Amphinemura	Plecoptera	Nemouridae	88 (7.4%)	1.23
*Anaciaeschna**	Odonata	Aeshnidae	1 (0.1%)	< 0.01
Anax	Odonata	Aeshnidae	29 (2.4%)	0.03
Ancylus	Gastropoda	Planorbidae	999 (83.6%)	40.5
Anodonta	Bivalvia	Unionidae	88 (7.4%)	0.09
Aphelocheirus	Heteroptera	Aphelocheiridae	503 (42.1%)	15.61
*Aplexa**	Gastropoda	Physidae	1 (0.1%)	< 0.01
*Arcynopteryx**	Plecoptera	Perlodidae	6 (0.5%)	0.01
*Arthroplea**	Ephemeroptera	Arthropleidae	1 (0.1%)	< 0.01
Asellidae	Isopoda	Asellidae	831 (69.5%)	63.13
Athripsodes	Trichoptera	Leptoceridae	743 (62.2%)	11.08
Atyaephyra	Decapoda	Atyidae	38 (3.2%)	1.37
*Aulonogyrus**	Coleoptera	Gyrinidae	3 (0.3%)	< 0.01
Austropotamobius	Decapoda	Astacidae	11 (0.9%)	0.03
Baetis	Ephemeroptera	Baetidae	1138 (95.2%)	262.05
*Belgrandia**	Gastropoda	Hydrobiidae	1 (0.1%)	0.33
Beraea	Trichoptera	Beraeidae	27 (2.3%)	0.02
*Beraeamyia**	Trichoptera	Beraeidae	2 (0.2%)	< 0.01
Beraeodes	Trichoptera	Beraeidae	187 (15.6%)	1.71
Bithynia	Gastropoda	Bithyniidae	452 (37.8%)	20.65
Boyeria	Odonata	Aeshnidae	259 (21.7%)	0.4
Brachycentrus	Trichoptera	Brachycentridae	381 (31.9%)	15.03
Brachycercus	Ephemeroptera	Caenidae	104 (8.7%)	0.27
Brachyptera	Plecoptera	Taeniopterygidae	44 (3.7%)	1.53

*Brachythemis**	Odonata	Libellulidae	2 (0.2%)	< 0.01
*Brachytron**	Odonata	Aeshnidae	2 (0.2%)	< 0.01
Brychius	Coleoptera	Haliplidae	33 (2.8%)	0.19
Bythinella	Gastropoda	Hydrobiidae	110 (9.2%)	0.95
Bythiospeum	Gastropoda	Hydrobiidae	25 (2.1%)	17.36
Caenis	Ephemeroptera	Caenidae	808 (67.6%)	54.29
Calamoceras	Trichoptera	Calamoceratidae	10 (0.8%)	0.05
Calopteryx	Odonata	Calopterygidae	1022 (85.5%)	15.04
*Capnia**	Plecoptera	Capniidae	3 (0.3%)	0.48
*Capnioneura**	Plecoptera	Capniidae	2 (0.2%)	< 0.01
*Capnopsis**	Plecoptera	Capniidae	2 (0.2%)	0.09
Centroptilum	Ephemeroptera	Baetidae	612 (51.2%)	9.48
Ceraclea	Trichoptera	Leptoceridae	313 (26.2%)	1.62
Chalcolestes	Odonata	Lestidae	32 (2.7%)	0.03
Cheumatopsyche	Trichoptera	Hydropsychidae	398 (33.3%)	46.43
Chimarra	Trichoptera	Philopotamidae	84 (7.0%)	1.7
Chloroperla	Plecoptera	Chloroperlidae	54 (4.5%)	0.18
Choroterpes	Ephemeroptera	Leptophlebiidae	42 (3.5%)	0.16
Cloeon	Ephemeroptera	Baetidae	352 (29.5%)	4.02
Corbicula	Bivalvia	Corbiculidae	198 (16.6%)	25.72
Cordulegaster	Odonata	Cordulegasteridae	238 (19.9%)	0.63
*Cordulia**	Odonata	Corduliidae	3 (0.3%)	< 0.01
Corophium	Amphipoda	Corophiidae	9 (0.8%)	1.08
Crangonyx	Amphipoda	Crangonyctidae	73 (6.1%)	3.28
Crunoecia	Trichoptera	Lepidostomatidae	18 (1.5%)	0.01
*Cymatia**	Heteroptera	Corixidae	2 (0.2%)	< 0.01
Cyphon	Coleoptera	Scirtidae	20 (1.7%)	0.1
Cyrnus	Trichoptera	Polycentropodidae	466 (39.0%)	4.03
*Dictyogenus**	Plecoptera	Perlodidae	3 (0.3%)	< 0.01
Dikerogammarus	Amphipoda	Pontogammaridae	46 (3.8%)	8.26
Dinocras	Plecoptera	Perlidae	107 (9.0%)	1.57
*Diplacodes**	Odonata	Libellulidae	1 (0.1%)	< 0.01
*Diplectrona**	Trichoptera	Hydropsychidae	6 (0.5%)	0.06
*Donacia**	Coleoptera	Chrysomelidae	1 (0.1%)	< 0.01
Dreissena	Bivalvia	Dreissenidae	9 (0.8%)	0.6
Dryops	Coleoptera	Dryopidae	275 (23.0%)	0.28
Dupophilus	Coleoptera	Elmidae	458 (38.3%)	20.24
Dytiscidae	Coleoptera	Dytiscidae	705 (59.0%)	5.38
Ecdyonurus	Ephemeroptera	Heptageniidae	527 (44.1%)	10.32
Echinogammarus	Amphipoda	Gammaridae	492 (41.2%)	525.51
Ecnomus	Trichoptera	Ecnomidae	86 (7.2%)	0.91

Electrogena	Ephemeroptera	Heptageniidae	155 (13.0%)	0.41
Elmis	Coleoptera	Elmidae	1036 (86.7%)	112.31
Epeorus	Ephemeroptera	Heptageniidae	271 (22.7%)	9.16
Ephemera	Ephemeroptera	Ephemeridae	937 (78.4%)	35.3
Ephemerella	Ephemeroptera	Ephemerellidae	944 (79.0%)	96.81
Ephoron	Ephemeroptera	Polymitarcyidae	114 (9.5%)	1.67
Epitheca	Odonata	Corduliidae	9 (0.8%)	0.01
*Erotesis**	Trichoptera	Leptoceridae	2 (0.2%)	< 0.01
Esolus	Coleoptera	Elmidae	877 (73.4%)	69.69
Euleuctra	Plecoptera	Leuctridae	328 (27.4%)	4.22
Ferrissia	Gastropoda	Planorbidae	207 (17.3%)	2.95
Galba	Gastropoda	Lymnaeidae	46 (3.8%)	0.08
Gammarus	Amphipoda	Gammaridae	1094 (91.5%)	725.43
Gerris	Heteroptera	Gerridae	599 (50.1%)	1.43
Glossosoma	Trichoptera	Glossosomatidae	180 (15.1%)	2.54
Goera	Trichoptera	Goeridae	428 (35.8%)	2.08
Gomphus	Odonata	Gomphidae	507 (42.4%)	2.67
Gyrinus	Coleoptera	Gyrinidae	100 (8.4%)	0.14
Habroleptoides	Ephemeroptera	Leptophlebiidae	176 (14.7%)	3.6
Habrophlebia	Ephemeroptera	Leptophlebiidae	384 (32.1%)	10.81
Haliplus	Coleoptera	Haliplidae	302 (25.3%)	1.47
Helodes	Coleoptera	Scirtidae	249 (20.8%)	0.93
Helophorus	Coleoptera	Helophoridae	261 (21.8%)	0.62
*Hemianax**	Odonata	Aeshnidae	1 (0.1%)	< 0.01
Heptagenia	Ephemeroptera	Heptageniidae	410 (34.3%)	5.77
Holocentropus	Trichoptera	Polycentropodidae	157 (13.1%)	0.71
Hydraena	Coleoptera	Hydraenidae	786 (65.8%)	16.31
Hydrochus	Coleoptera	Hydrochidae	88 (7.4%)	0.1
Hydrocyphon	Coleoptera	Scirtidae	96 (8.0%)	0.59
Hydrometra	Heteroptera	Hydrometridae	230 (19.2%)	0.28
Hydrophilidae	Coleoptera	Hydrophilidae	386 (32.3%)	0.6
Hydropsyche	Trichoptera	Hydropsychidae	1054 (88.2%)	203.89
Hydroptila	Trichoptera	Hydroptilidae	708 (59.2%)	16.5
*Hydroscapha**	Coleoptera	Hydroscaphidae	2 (0.2%)	< 0.01
*Hygrobia**	Coleoptera	Hygrobiidae	2 (0.2%)	0.01
*Isogenus**	Plecoptera	Perlodidae	3 (0.3%)	0.01
Isoperla	Plecoptera	Perlodidae	260 (21.8%)	4.38
Ithytrichia	Trichoptera	Hydroptilidae	440 (36.8%)	19.62
Lasiocephala	Trichoptera	Lepidostomatidae	146 (12.2%)	4.58
Lepidostoma	Trichoptera	Lepidostomatidae	456 (38.2%)	12.93
Leptocerus	Trichoptera	Leptoceridae	138 (11.5%)	2.72

Leptophlebia	Ephemeroptera	Leptophlebiidae	16 (1.3%)	0.03
*Lestes**	Odonata	Lestidae	3 (0.3%)	< 0.01
*Leucorrhinia**	Odonata	Libellulidae	2 (0.2%)	< 0.01
Leuctra	Plecoptera	Leuctridae	721 (60.3%)	60.35
Libellula	Odonata	Libellulidae	42 (3.5%)	0.07
Limnebius	Coleoptera	Hydraenidae	92 (7.7%)	0.21
Limnephilidae	Trichoptera	Limnephilidae	936 (78.3%)	20.39
Limnius	Coleoptera	Elmidae	935 (78.2%)	41.57
Lithax	Trichoptera	Goeridae	31 (2.6%)	0.5
*Lithoglyphus**	Gastropoda	Hydrobiidae	3 (0.3%)	< 0.01
Lymnaea	Gastropoda	Lymnaeidae	19 (1.6%)	0.03
Lype	Trichoptera	Psychomyiidae	532 (44.5%)	1.51
*Macromia**	Odonata	Corduliidae	1 (0.1%)	< 0.01
Macronychus	Coleoptera	Elmidae	180 (15.1%)	0.4
*Macroplea**	Coleoptera	Chrysomelidae	1 (0.1%)	< 0.01
*Margaritifera**	Bivalvia	Margaritiferidae	4 (0.3%)	< 0.01
*Marthamea**	Plecoptera	Perlidae	1 (0.1%)	< 0.01
Mesovelia	Heteroptera	Mesoveliidae	39 (3.3%)	0.06
Metalype	Trichoptera	Psychomyiidae	15 (1.3%)	0.02
Micrasema	Trichoptera	Brachycentridae	274 (22.9%)	21.5
Micronecta	Heteroptera	Corixidae	684 (57.2%)	42.47
Molanna	Trichoptera	Molannidae	105 (8.8%)	0.32
Molannodes	Trichoptera	Molannidae	13 (1.1%)	0.02
Mystacides	Trichoptera	Leptoceridae	830 (69.5%)	12.34
Myxas	Gastropoda	Lymnaeidae	10 (0.8%)	0.02
Naucoridae	Heteroptera	Naucoridae	54 (4.5%)	1.19
Nemoura	Plecoptera	Nemouridae	353 (29.5%)	10.43
*Nemurella**	Plecoptera	Nemouridae	2 (0.2%)	< 0.01
*Neoephemera**	Ephemeroptera	Neoephemeridae	1 (0.1%)	< 0.01
Nepidae	Heteroptera	Nepidae	178 (14.9%)	0.19
Neureclipsis	Trichoptera	Polycentropodidae	130 (10.9%)	3.03
Niphargus	Amphipoda	Niphargidae	46 (3.8%)	0.06
Normandia	Coleoptera	Elmidae	111 (9.3%)	2.19
*Noterus**	Coleoptera	Noteridae	4 (0.3%)	< 0.01
Notidobia	Trichoptera	Sericostomatidae	254 (21.3%)	1.07
Notonectidae	Heteroptera	Notonectidae	190 (15.9%)	0.24
Ochthebius	Coleoptera	Hydraenidae	76 (6.4%)	0.09
Odontocerum	Trichoptera	Odontoceridae	148 (12.4%)	2.55
Oecetis	Trichoptera	Leptoceridae	520 (43.5%)	5.02
Oecismus	Trichoptera	Sericostomatidae	10 (0.8%)	0.01
Oligoneuriella	Ephemeroptera	Oligoneuriidae	75 (6.3%)	1.59

Oligoplectrum	Trichoptera	Brachycentridae	124 (10.4%)	46.94
Onychogomphus	Odonata	Gomphidae	424 (35.5%)	1.98
Ophiogomphus	Odonata	Gomphidae	26 (2.2%)	0.05
Orconectes	Decapoda	Cambaridae	275 (23.0%)	0.42
Orectochilus	Coleoptera	Gyrinidae	524 (43.8%)	3.62
Orthetrum	Odonata	Libellulidae	26 (2.2%)	0.04
Orthotrichia	Trichoptera	Hydroptilidae	108 (9.0%)	3.24
Oulimnius	Coleoptera	Elmidae	1003 (83.9%)	70.38
Oxyethira	Trichoptera	Hydroptilidae	17 (1.4%)	0.02
Oxygastra	Odonata	Corduliidae	18 (1.5%)	0.05
Pacifastacus	Decapoda	Astacidae	215 (18.0%)	1.7
*Paragomphus**	Odonata	Gomphidae	2 (0.2%)	< 0.01
Paraleptophlebia	Ephemeroptera	Leptophlebiidae	551 (46.1%)	10.03
Peltodytes	Coleoptera	Haliplidae	11 (0.9%)	0.01
Perla	Plecoptera	Perlidae	189 (15.8%)	1.85
Perlodes	Plecoptera	Perlodidae	180 (15.1%)	1.14
Philopotamus	Trichoptera	Philopotamidae	89 (7.4%)	1.13
Phryganea	Trichoptera	Phryganeidae	18 (1.5%)	0.01
Physa	Gastropoda	Physidae	374 (31.3%)	8.3
Physella	Gastropoda	Physidae	247 (20.7%)	4.72
Pisidium	Bivalvia	Sphaeriidae	1058 (88.5%)	151.98
Platycnemis	Odonata	Platycnemididae	624 (52.2%)	9.16
Plea	Heteroptera	Pleidae	35 (2.9%)	0.23
Plectrocnemia	Trichoptera	Polycentropodidae	93 (7.8%)	0.32
Polycentropus	Trichoptera	Polycentropodidae	804 (67.3%)	20.68
Pomatinus	Coleoptera	Dryopidae	169 (14.1%)	0.24
Potamanthus	Ephemeroptera	Potamanthidae	145 (12.1%)	4.95
Potamophilus	Coleoptera	Elmidae	91 (7.6%)	0.3
Potamopyrgus	Gastropoda	Hydrobiidae	811 (67.9%)	491.73
Potomida	Bivalvia	Unionidae	9 (0.8%)	0.01
Procambarus	Decapoda	Cambaridae	10 (0.8%)	0.01
Procloeon	Ephemeroptera	Baetidae	513 (42.9%)	5.18
Protonemura	Plecoptera	Nemouridae	320 (26.8%)	34.48
Pseudanodonta	Bivalvia	Unionidae	15 (1.3%)	0.01
Pseudocentroptilum	Ephemeroptera	Baetidae	125 (10.5%)	0.4
*Pseudoneureclipsis**	Trichoptera	Ecnomidae	1 (0.1%)	< 0.01
Psychomyia	Trichoptera	Psychomyiidae	459 (38.4%)	13.04
*Ptilocolepus**	Trichoptera	Hydroptilidae	5 (0.4%)	0.01
Radix	Gastropoda	Lymnaeidae	562 (47.0%)	7.12
Raptobaetopus	Ephemeroptera	Baetidae	34 (2.8%)	0.12
*Rhabdiopteryx**	Plecoptera	Taeniopterygidae	4 (0.3%)	0.03

Rhithrogena	Ephemeroptera	Heptageniidae	325 (27.2%)	9.33
Rhyacophila	Trichoptera	Rhyacophilidae	803 (67.2%)	16.51
Riolus	Coleoptera	Elmidae	271 (22.7%)	9.64
*Schizopelex**	Trichoptera	Sericostomatidae	1 (0.1%)	< 0.01
*Scirtes**	Coleoptera	Scirtidae	5 (0.4%)	0.01
Sericostoma	Trichoptera	Sericostomatidae	485 (40.6%)	17.14
Setodes	Trichoptera	Leptoceridae	108 (9.0%)	1.01
Silo	Trichoptera	Goeridae	486 (40.7%)	8.96
Siphlonurus	Ephemeroptera	Siphlonuridae	17 (1.4%)	0.07
Siphonoperla	Plecoptera	Chloroperlidae	164 (13.7%)	1.03
Somatochlora	Odonata	Corduliidae	33 (2.8%)	0.03
Sphaerium	Bivalvia	Sphaeriidae	624 (52.2%)	43.52
*Stactobia**	Trichoptera	Hydroptilidae	1 (0.1%)	< 0.01
*Stactobiella**	Trichoptera	Hydroptilidae	1 (0.1%)	< 0.01
Stagnicola	Gastropoda	Lymnaeidae	36 (3.0%)	0.08
Stenelmis	Coleoptera	Elmidae	315 (26.4%)	3.3
Sympecma	Odonata	Lestidae	7 (0.6%)	0.01
Sympetrum	Odonata	Libellulidae	8 (0.7%)	< 0.01
Synagapetus	Trichoptera	Glossosomatidae	12 (1.0%)	0.19
Taeniopteryx	Plecoptera	Taeniopterygidae	146 (12.2%)	2.09
Theodoxus	Gastropoda	Neritidae	236 (19.7%)	24.89
Thraulus	Ephemeroptera	Leptophlebiidae	10 (0.8%)	0.02
Thremma	Trichoptera	Uenoidae	13 (1.1%)	0.25
Tinodes	Trichoptera	Psychomyiidae	302 (25.3%)	1.81
Torleya	Ephemeroptera	Ephemerellidae	147 (12.3%)	1.37
Triaenodes	Trichoptera	Leptoceridae	33 (2.8%)	0.1
*Trichostegia**	Trichoptera	Phryganeidae	3 (0.3%)	< 0.01
Unio	Bivalvia	Unionidae	97 (8.1%)	0.08
Valvata	Gastropoda	Valvatidae	178 (14.9%)	2.48
Viviparus	Gastropoda	Viviparidae	74 (6.2%)	4.88
Wormaldia	Trichoptera	Philopotamidae	46 (3.8%)	0.17
Xanthoperla	Plecoptera	Chloroperlidae	10 (0.8%)	0.15
Ylodes	Trichoptera	Leptoceridae	34 (2.8%)	0.08
