# Supplementary material for: Direct habitat descriptors improve the understanding of the organization of fish and macroinvertebrate communities across a large catchment
Source: PLoS One. 2022 Sep 22;17(9):e0274167. doi: 10.1371/journal.pone.0274167 (PMC9498974; doi:10.1371/journal.pone.0274167)
Supplement: S3 Table — (PDF) [file pone.0274167.s004.pdf]

S3 Table. Position of the centroids of the 43 fish species on the three factorial axes of the NMDS performed on the fish dataset.

Scientific name	Axis 1	Axis 2	Axis 3
Abramis brama	-1.27	0.66	0.25
Alburnoides bipunctatus	-0.67	0.37	-0.76
Alburnus alburnus	-1.09	0.68	-0.40
Ambloplites rupestris	-1.41	1.23	-0.52
Ameiurus melas	-1.06	-0.27	0.16
Anguilla anguilla	-0.93	0.27	0.29
Aspius aspius	-1.48	1.31	-0.15
Barbatula barbatula	0.05	-0.78	-0.02
Barbus barbus	-0.74	0.42	-0.82
Blicca bjoerkna	-1.35	0.91	0.16
Carassius sp.	-1.06	-0.26	0.43
Chondrostoma nasus	-1.02	0.79	-0.74
Cobitis taenia	-1.44	1.16	0.00
Cottus gobio	0.44	-0.05	1.02
Cyprinus carpio	-1.00	-0.37	0.26
Esox lucius	-1.02	0.18	0.62
Gasterosteus gymnurus	-1.07	-0.51	0.11
Gobio gobio	-0.46	-0.20	-0.46
Gymnocephalus cernuus	-1.18	0.95	-0.13
Lampetra planeri	0.23	-0.20	0.52
Lepomis gibbosus	-0.94	-0.52	-0.04
Leucaspius delineatus	-1.06	0.23	0.35
Leuciscus idus	-1.57	1.07	0.30
Leuciscus leuciscus	-0.69	0.37	-0.47
Liza ramada	-1.44	1.40	-0.29
Lota lota	-0.75	0.73	-0.30
Micropterus salmoides	-1.31	0.02	0.32
Parachondrostoma toxostoma	-0.83	0.54	-1.30
Perca fluviatilis	-0.78	-0.02	0.25
Petromyzon marinus	-1.14	1.16	-0.57
Phoxinus phoxinus	0.37	-1.04	-0.36
Pseudorasbora parva	-1.15	-0.06	-0.21
Pungitius laevis	-0.49	-0.75	1.42
Rhodeus amarus	-1.23	0.63	-0.20
Rutilus rutilus	-0.92	0.00	0.08
Salmo salar	0.30	0.61	-0.02
Salmo trutta	1.53	0.34	-0.10
Sander luciopercia	-1.30	0.74	0.01
Scardinius erythrophthalmus	-1.13	-0.22	0.15

Silurus glanis	-1.38	1.11	-0.17
Squalius cephalus	-0.63	-0.01	-0.36
Thymallus thymallus	0.06	0.18	-0.19
Tinca tinca	-0.97	0.10	0.21
