# Supplementary material for: Direct habitat descriptors improve the understanding of the organization of fish and macroinvertebrate communities across a large catchment
Source: PLoS One. 2022 Sep 22;17(9):e0274167. doi: 10.1371/journal.pone.0274167 (PMC9498974; doi:10.1371/journal.pone.0274167)
Supplement: S4 Table — (PDF) [file pone.0274167.s005.pdf]

S4 Table. Position of the centroids of the 186 macroinvertebrates taxa on the three factorial axes of the NMDS performed on the macroinvertebrate dataset.

Macroinvertebrates' genus	Axis 1	Axis 2	Axis 3
Acentrella	-0.60	0.40	0.49
Acroloxus	0.73	0.00	-0.45
Adicella	-0.66	-0.05	-0.34
Aeshna	0.13	-0.50	0.20
Agapetus	-0.16	-0.45	-0.74
Agraylea	0.51	0.73	0.16
Allotrichia	0.42	0.72	0.50
Amphinemura	-1.00	-0.40	0.17
Anax	1.08	1.06	0.01
Ancylus	-0.08	-0.15	0.23
Anodonta	0.85	0.05	-0.04
Aphelocheirus	0.07	0.52	0.22
Asellidae	0.55	-0.56	0.40
Athripsodes	0.20	0.11	-0.11
Atyaephyra	1.21	0.91	0.02
Austropotamobius	-0.73	-0.65	-0.28
Baetis	-0.17	-0.13	0.27
Beraea	-0.81	-0.61	0.12
Beraeodes	0.28	-0.30	-0.57
Bithynia	0.80	-0.24	0.10
Boyeria	-0.07	0.34	-0.24
Brachycentrus	-0.19	0.69	0.09
Brachycercus	-0.10	0.57	0.64
Brachyptera	-0.97	-0.31	0.36
Brychius	0.07	0.01	-1.28
Bythinella	-0.80	-0.51	0.00
Bythiospeum	0.49	-0.01	-0.95
Caenis	0.39	0.51	0.12
Calamoceras	-0.08	0.40	-0.83
Calopteryx	0.08	0.00	0.08
Centroptilum	0.25	0.33	-0.17
Ceraclea	0.37	0.65	0.09
Chalcolestes	1.07	-0.11	0.18
Cheumatopsyche	-0.16	0.69	0.23
Chimarra	-0.29	0.88	0.16
Chloroperla	-1.15	0.05	-0.10
Choroterpes	0.18	0.99	0.84
Cloeon	1.10	-0.22	0.34
Corbicula	0.61	1.10	0.30
Cordulegaster	-0.72	-0.55	-0.03
Corophium	0.56	1.68	0.58
Crangonyx	0.59	0.87	0.56

Crunoecia	-1.21	-0.44	-0.31
Cyphon	-0.86	0.26	-0.39
Cyrnus	0.34	0.24	0.11
Dikerogammarus	0.64	1.46	0.56
Dinocras	-1.25	-0.15	0.00
Dreissena	1.75	1.08	0.12
Dryops	-0.05	-0.35	0.09
Dupophilus	-0.82	0.09	0.01
Dytiscidae	-0.06	-0.37	0.08
Ecdyonurus	-0.65	0.03	0.40
Echinogammarus	0.28	0.04	-0.90
Ecnomus	1.33	0.98	0.31
Electrogena	-0.21	0.25	0.48
Elmis	-0.28	-0.14	-0.37
Epeorus	-1.04	-0.03	0.10
Ephemera	-0.09	-0.24	-0.01
Ephemerella	-0.28	-0.09	0.33
Ephoron	0.35	0.92	0.77
Epitheca	0.21	0.56	0.21
Esolus	-0.14	0.42	-0.17
Euleuctra	-0.25	0.44	-0.10
Ferrissia	0.88	0.57	0.58
Galba	0.27	-0.38	0.05
Gammarus	0.19	-0.82	-0.12
Gerris	0.23	-0.07	0.26
Glossosoma	-1.01	-0.07	0.01
Goera	0.18	0.03	-0.07
Gomphus	0.01	0.53	0.39
Gyrinus	0.37	-0.32	-0.35
Habroleptoides	-0.92	-0.02	0.23
Habrophlebia	-0.61	-0.37	0.39
Haliplus	0.85	-0.12	-0.29
Helodes	-0.27	-0.83	-0.61
Helophorus	0.26	-0.69	0.01
Heptagenia	-0.03	0.63	0.56
Holocentropus	0.02	0.14	0.17
Hydraena	-0.56	-0.22	0.05
Hydrochus	0.34	0.06	0.05
Hydrocyphon	-1.01	0.04	0.13
Hydrometra	0.36	-0.27	0.27
Hydrophilidae	0.32	-0.05	0.29
Hydropsyche	-0.06	0.11	0.20
Hydroptila	0.25	0.22	0.10
Isoperla	-0.97	-0.34	0.42
Ithytrichia	-0.12	0.40	-0.43
Lasiocephala	-0.47	0.08	-0.71

Lepidostoma	-0.22	0.28	-0.58
Leptocerus	0.41	0.80	-0.21
Leptophlebia	-0.39	0.18	-0.05
Leuctra	-0.62	0.06	0.25
Libellula	1.02	-0.35	0.45
Limnebius	-0.91	-0.14	-0.03
Limnephilidae	-0.40	-0.53	-0.11
Limnius	-0.24	0.01	-0.45
Lithax	-0.69	0.16	0.01
Lymnaea	0.65	0.28	-0.41
Lype	0.11	-0.04	-0.39
Macronychus	0.17	0.86	-0.27
Mesovelia	0.24	0.09	-0.13
Metalype	0.47	0.18	0.20
Micrasema	-0.99	0.03	0.04
Micronecta	0.43	0.18	0.35
Molanna	0.62	0.30	-0.82
Molannodes	0.62	0.50	-0.87
Mystacides	0.12	0.30	-0.02
Myxas	0.41	-0.59	-0.11
Naucoridae	0.61	-0.40	1.98
Nemoura	-0.73	-0.21	0.60
Nepidae	0.35	-0.78	0.09
Neureclipsis	0.90	0.44	0.54
Niphargus	-0.11	-0.10	-0.31
Normandia	0.22	0.77	-0.63
Notidobia	0.20	-0.27	-0.68
Notonectidae	0.53	-0.64	0.26
Ochthebius	0.37	-0.54	-0.30
Odontocerum	-1.09	-0.41	-0.26
Oecetis	-0.12	0.64	0.02
Oecismus	-1.07	0.32	-0.37
Oligoneuriella	-0.42	0.64	0.68
Oligoplectrum	-0.49	0.72	0.18
Onychogomphus	-0.15	0.63	0.25
Ophiogomphus	-0.02	0.97	0.20
Orconectes	0.52	0.54	0.17
Orectochilus	-0.24	0.34	-0.07
Orthetrum	0.89	0.24	-0.33
Orthotrichia	0.74	1.15	0.11
Oulimnius	0.09	0.08	-0.25
Oxyethira	-0.41	0.36	-0.43
Oxygastra	0.24	1.16	-0.13
Pacifastacus	-0.40	0.03	0.49
Paraleptophlebia	-0.38	0.21	0.29
Peltodytes	0.79	0.04	-0.03

Perla	-1.00	0.02	0.13
Perlodes	-1.01	-0.01	0.15
Philopotamus	-1.27	-0.35	-0.04
Phryganea	0.99	0.03	-0.43
Physa	0.82	0.48	0.17
Physella	0.93	-0.04	0.40
Pisidium	0.43	-0.68	-0.43
Platycnemis	0.63	0.34	0.33
Plea	0.54	-1.35	0.14
Plectrocnemia	-0.64	-0.71	-0.06
Polycentropus	-0.10	0.17	0.01
Pomatinus	-0.08	0.23	-0.21
Potamanthus	0.28	0.61	1.07
Potamophilus	0.28	1.25	0.22
Potamopyrgus	0.37	-0.24	-0.46
Potomida	0.66	1.39	-0.40
Procambarus	0.99	0.40	0.23
Procloeon	0.28	0.61	0.32
Protonemura	-1.12	-0.23	0.10
Pseudanodonta	0.49	0.41	-0.52
Pseudocentroptilum	-0.41	0.30	0.25
Psychomyia	-0.11	0.62	0.48
Radix	0.55	-0.21	-0.03
Raptobaetopus	0.48	1.44	0.49
Rhithrogena	-0.93	-0.20	0.15
Rhyacophila	-0.50	-0.12	0.16
Riolus	0.20	0.14	-0.97
Sericostoma	-0.60	-0.20	-0.49
Setodes	-0.03	0.87	0.30
Silo	-0.48	-0.15	-0.33
Siphlonurus	-0.57	-0.35	0.95
Siphonoperla	-1.09	-0.16	0.23
Somatochlora	0.55	-0.19	0.06
Sphaerium	0.66	-0.35	-0.30
Stagnicola	0.97	-1.41	0.40
Stenelmis	0.06	0.72	-0.33
Sympecma	0.46	0.05	0.51
Sympetrum	0.92	0.24	0.25
Synagapetus	-0.08	-0.03	1.36
Taeniopteryx	-0.68	0.37	0.06
Theodoxus	0.37	0.49	-0.53
Thraulus	-0.22	0.95	0.12
Thremma	-1.62	-0.42	-0.05
Tinodes	0.27	-0.05	0.14
Torleya	-0.74	0.37	-0.14
Triaenodes	-0.15	0.90	-0.36

Unio	0.35	0.35	-0.43
Valvata	0.86	-0.33	-0.16
Viviparus	-0.31	-1.84	1.57
Wormaldia	-1.03	0.15	-0.13
Xanthoperla	0.44	1.47	0.58
Ylodes	0.05	0.96	0.01
