# Supplementary material for: Direct habitat descriptors improve the understanding of the organization of fish and macroinvertebrate communities across a large catchment
Source: PLoS One. 2022 Sep 22;17(9):e0274167. doi: 10.1371/journal.pone.0274167 (PMC9498974; doi:10.1371/journal.pone.0274167)
Supplement: S5 Table — (PDF) [file pone.0274167.s006.pdf]

S5 Table. Position of the centroids of the 43 fish species (bold) and 170 macroinvertebrates taxa (non-bold) on the three factorial axes of the NMDS performed on the common dataset.

Taxa name	Axis 1	Axis 2	Axis 3
Abramis brama	0.88	-0.01	-0.13
Alburnoides bipunctatus	0.29	0.57	-0.39
Alburnus alburnus	0.73	0.46	-0.21
Ambloplites rupestris	0.96	1.08	-0.12
Ameiurus melas	0.92	-0.39	-0.55
Anguilla anguilla	0.72	-0.12	0.06
Aspius aspius	1.27	0.69	-0.17
Barbatula barbatula	-0.06	-0.44	-0.23
Barbus barbus	0.41	0.55	-0.22
Blicca bjoerkna	0.99	0.27	-0.01
Carassius sp.	0.61	-0.57	-0.49
Chondrostoma nasus	0.60	0.71	-0.34
Cobitis taenia	1.14	0.41	-0.19
Cottus gobio	-0.32	-0.37	0.33
Cyprinus carpio	0.58	-0.33	-0.45
Esox lucius	0.70	-0.15	0.11
Gasterosteus gymnurus	0.35	-0.76	-1.28
Gobio gobio	0.29	0.03	-0.43
Gymnocephalus cernuus	0.81	0.37	0.05
Lampetra planeri	-0.28	-0.04	0.07
Lepomis gibbosus	0.61	-0.33	-0.56
Leucaspius delineatus	0.74	-0.34	-0.15
Leuciscus idus	1.28	0.10	-0.25
Leuciscus leuciscus	0.44	0.41	-0.21
Liza ramada	1.39	0.94	-0.33
Lota lota	0.22	0.18	-0.02
Micropterus salmoides	0.89	-0.10	-0.01
Parachondrostoma toxostoma	0.55	0.28	-0.33
Perca fluviatilis	0.44	-0.18	-0.21
Petromyzon marinus	0.72	0.93	-0.21
Phoxinus phoxinus	-0.21	-0.51	0.01
Pseudorasbora parva	0.74	-0.19	-0.59
Pungitius laevis	0.20	-1.04	0.65
Rhodeus amarus	0.90	0.18	-0.25
Rutilus rutilus	0.66	-0.25	-0.23
Salmo salar	-0.56	-0.27	1.27
Salmo trutta	-1.02	-0.19	-0.37
Sander luciopercia	0.87	0.27	-0.10
Scardinius erythrophthalmus	0.65	-0.40	-0.54
Silurus glanis	0.98	0.66	0.01

Squalius cephalus	0.34	0.07	-0.33
Thymallus thymallus	-0.52	0.63	-0.14
Tinca tinca	0.63	-0.31	-0.28
Acentrella	-0.81	0.32	-0.27
Acroloxus	0.66	-0.51	0.46
Adicella	-0.77	0.23	0.28
Agapetus	-0.48	-0.44	0.37
Agraylea	0.60	0.45	0.31
Amphinemura	-1.37	0.04	-0.23
Anax	1.19	0.25	0.45
Ancylus	-0.14	-0.08	0.00
Anodonta	0.73	-0.12	0.20
Aphelocheirus	0.25	0.38	-0.01
Athripsodes	0.23	-0.11	0.15
Atyaephyra	1.34	0.18	0.21
Baetis	-0.27	0.01	-0.21
Beraea	-0.75	0.02	-0.20
Beraeodes	0.05	-0.65	0.24
Bithynia	0.64	-0.16	0.26
Boyeria	-0.07	0.14	0.22
Brachycentrus	-0.06	0.53	0.13
Brachycercus	0.10	0.34	-0.48
Brachyptera	-1.12	-0.27	-0.52
Brychius	-0.19	-0.61	0.99
Bythinella	-0.88	-0.08	0.19
Bythiospeum	0.27	-0.65	1.13
Caenis	0.43	0.17	0.17
Calopteryx	0.03	-0.06	-0.11
Centroptilum	0.26	-0.08	0.30
Ceraclea	0.47	0.31	0.18
Chalcolestes	0.73	-0.68	0.83
Cheumatopsyche	-0.02	0.53	0.01
Chimarra	-0.05	0.77	0.22
Chloroperla	-1.16	0.35	-0.03
Choroterpes	0.47	0.82	0.01
Cloeon	0.84	-0.36	-0.16
Corbicula	0.86	0.71	0.16
Cordulegaster	-0.99	-0.32	-0.20
Corophium	1.03	1.09	-0.12
Crangonyx	0.73	0.48	-0.11
Crunoecia	-1.43	0.29	0.11
Cyphon	-1.19	0.49	0.26
Cyrnus	0.32	-0.02	-0.01
Dikerogammarus	1.23	0.96	-0.22
Dinocras	-1.29	0.46	0.18

Dreissena	1.69	0.08	0.26
Dryops	-0.20	-0.20	-0.30
Dupophilus	-0.84	0.22	0.02
Ecdyonurus	-0.69	0.29	-0.25
Echinogammarus	0.25	-0.35	0.70
Ecnomus	1.35	0.22	0.14
Electrogena	-0.26	0.21	-0.44
Elmis	-0.42	-0.18	0.26
Epeorus	-1.15	0.41	-0.01
Ephemera	-0.17	-0.22	0.15
Ephemerella	-0.32	0.05	-0.03
Ephoron	0.59	0.78	-0.02
Epitheca	0.39	0.08	-0.48
Esolus	0.00	0.23	0.31
Euleuctra	-0.16	0.35	0.24
Ferrissia	0.94	0.07	0.02
Galba	0.21	-0.47	0.30
Gammarus	-0.15	-0.51	-0.26
Gerris	0.02	-0.22	-0.16
Glossosoma	-1.16	0.29	0.18
Goera	0.07	-0.23	0.09
Gomphus	0.15	0.39	-0.18
Gyrinus	0.21	-0.55	0.17
Habroleptoides	-0.97	0.29	-0.19
Habrophlebia	-0.84	-0.10	-0.42
Haliplus	0.64	-0.55	0.27
Helodes	-0.66	-0.74	-0.13
Helophorus	-0.05	-0.54	-0.31
Heptagenia	0.15	0.52	-0.28
Holocentropus	-0.18	0.21	0.09
Hydraena	-0.71	0.00	-0.15
Hydrochus	0.04	-0.04	-0.24
Hydrocyphon	-1.02	0.32	-0.08
Hydrometra	0.12	-0.21	-0.31
Hydropsyche	-0.09	0.13	-0.11
Hydroptila	0.23	-0.08	-0.16
Isoperla	-1.14	0.15	-0.15
Ithytrichia	-0.08	0.11	0.32
Lasiocephala	-0.62	-0.04	0.53
Lepidostoma	-0.18	0.03	0.62
Leptocerus	0.57	0.29	0.55
Leptophlebia	-0.63	0.09	0.22
Leuctra	-0.66	0.25	-0.17
Libellula	0.48	-1.25	0.14
Limnebius	-1.01	0.26	0.12

Limnius	-0.30	-0.10	0.30
Lithax	-0.84	0.24	-0.20
Lymnaea	0.74	-0.39	0.90
Lype	0.09	-0.28	0.25
Macronychus	0.38	0.41	0.31
Mesovelia	0.11	-0.38	-0.05
Metalype	-0.06	-0.07	0.43
Micrasema	-1.04	0.44	0.01
Micronecta	0.31	0.12	-0.17
Molanna	0.52	-0.60	0.74
Molannodes	0.66	-0.48	0.81
Mystacides	0.14	0.04	0.16
Nemoura	-0.87	0.07	-0.47
Neureclipsis	0.98	0.06	-0.35
Niphargus	-0.15	-0.11	0.46
Normandia	0.36	0.28	0.84
Notidobia	0.05	-0.48	0.40
Ochthebius	-0.29	-0.21	-0.15
Odontocerum	-1.31	0.16	0.26
Oecetis	0.08	0.44	0.20
Oecismus	-1.02	0.37	0.47
Oligoneuriella	-0.29	0.89	0.06
Oligoplectrum	-0.26	0.78	0.01
Onychogomphus	0.07	0.47	-0.01
Ophiogomphus	0.24	0.42	0.08
Orconectes	0.48	0.18	-0.05
Orectochilus	-0.14	0.26	0.06
Orthetrum	1.03	0.33	0.17
Orthotrichia	0.92	0.59	0.34
Oulimnius	0.07	-0.13	0.20
Oxyethira	-0.48	-0.02	0.46
Oxygastra	0.54	0.65	0.39
Pacifastacus	-0.47	0.14	-0.71
Paraleptophlebia	-0.39	0.16	-0.33
Peltodytes	0.60	0.27	0.33
Perla	-1.00	0.45	-0.04
Perlodes	-1.09	0.42	0.01
Philopotamus	-1.53	0.20	0.02
Phryganea	0.83	-0.55	0.16
Physa	0.79	0.01	0.02
Physella	0.73	-0.63	-0.54
Pisidium	0.19	-0.64	0.16
Platycnemis	0.57	0.08	0.06
Plea	0.45	-1.43	-0.92
Plectrocnemia	-0.88	-0.46	-0.42

Polycentropus	-0.06	0.05	-0.01
Pomatinus	-0.04	0.15	0.15
Potamanthus	0.56	0.79	0.04
Potamophilus	0.69	0.84	0.20
Potamopyrgus	0.27	-0.48	0.23
Potomida	0.93	0.61	0.34
Procloeon	0.43	0.34	-0.01
Protonemura	-1.33	0.18	-0.18
Pseudanodonta	0.32	-0.06	0.39
Pseudocentroptilum	-0.32	0.28	-0.25
Psychomyia	0.11	0.59	-0.19
Radix	0.34	-0.31	0.35
Raptobaetopus	0.73	0.97	0.09
Rhithrogena	-1.08	0.15	-0.14
Rhyacophila	-0.59	0.06	-0.11
Riolus	0.23	-0.47	0.75
Sericostoma	-0.78	-0.20	0.34
Setodes	0.26	0.69	0.03
Silo	-0.59	-0.22	0.17
Siphlonurus	-0.73	-0.59	-0.67
Siphonoperla	-1.26	0.31	-0.14
Somatochlora	0.43	-0.27	-0.49
Sphaerium	0.42	-0.46	0.16
Stagnicola	0.27	-1.00	0.98
Stenelmis	0.19	0.36	0.49
Taeniopteryx	-0.56	0.57	0.11
Theodoxus	0.36	-0.03	0.80
Thraulus	-0.19	0.80	0.32
Thremma	-1.91	0.45	0.02
Tinodes	0.29	-0.39	0.09
Torleya	-0.73	0.39	0.16
Triaenodes	0.37	0.60	0.41
Unio	0.40	-0.06	0.25
Valvata	0.46	-0.17	0.50
Viviparus	-0.36	-1.89	3.03
Wormaldia	-1.06	0.27	0.17
Xanthoperla	0.76	1.07	0.04
Ylodes	0.24	0.60	0.39
