# Supplementary material for: Direct habitat descriptors improve the understanding of the organization of fish and macroinvertebrate communities across a large catchment
Source: PLoS One. 2022 Sep 22;17(9):e0274167. doi: 10.1371/journal.pone.0274167 (PMC9498974; doi:10.1371/journal.pone.0274167)
Supplement: S1 Appendix — (PDF) [file pone.0274167.s007.pdf]

S1 Appendix. Monte Carlo cross-validation procedure and sensitivity analysis of RDA to uncertainties in environmental variables derived from physical-based models.

Monte-Carlo cross-validation procedure and RDA's predictive power

In complement to the results regarding the explanatory power of the RDA, we also tested their predictive power on the remaining 30% of sub-basins, which were not considered in the calibration of the RDA (Table 1).

Similar to the results for the explanatory power of the RDA, all direct variables (i.e. hydraulic variables, water temperature, and both combined) showed a higher predictive R^2 on average than their surrogate counterparts (i.e. discharge, air temperature, and both combined) for all three biological groups, except for discharge vs. hydraulic variables for the common dataset for which the difference was slightly opposite. For macroinvertebrate, differences in predictive power between water temperature and air temperature and between combined direct variables and combined surrogate variables were not significant, as for common dataset except for the difference between water temperature and air temperature (i.e. the mean of the surrogate-R^2 is not included in the confidence interval of the direct-R^2 but the confidence intervals overlap), with direct variables predicting biological ordinations better than surrogate variables. The main difference with the results regarding explanatory power was the wider confidence intervals around the R^2, which made the difference between pairs of environmental variables non-significant due to overlapping confidence intervals. Moreover, predictive R^2 were higher than their explanatory counterparts. This highlighted the predominant role of environmental gradients in structuring the spatial organization of aquatic communities at the catchment scale.

Table 1. Importance of the environmental variables in explaining or predicting the biological ordination of fish (A), macroinvertebrates (B) and common (C) datasets: average coefficients of determination (R^2) and associated 95% confidence intervals derived from RDA. Explanatory R^2 was derived from RDA performed on reaches contained in 70% of sub-basins; predictive R^2 was derived from predictive RDA performed on reaches contained in the remaining 30% of sub-basins.

**Table A** ***Fish***	**Environmental variables**	**Explanatory R^2**	**Predictive R^2**
**Hydrological regime**	Discharge	28.1 [25.4; 30.9]	52.4 [43.2; 61.6]
	Hydraulic variables	43.9 [41.3; 46.5]	58.7 [53.0; 64.4]
**Thermal regime**	Air temperature	41.0 [37.6; 44.4]	54.1 [44.9; 63.3]
	Water temperature	47.1 [43.7; 50.5]	58.2 [49.4; 67.0]
**Surrogate variables**	Air temperature + discharge	51.8 [49.1; 54.6]	62.3 [55.5; 69.1]
**Direct variables**	Water temperature + hydraulic variables	54.7 [52.1; 57.4]	64.5 [58.3; 70.7]

**Table B** *Macroinv.*	**Environmental variables**	**Explanatory R^2**	**Predictive R^2**
**Hydrological regime**	Discharge	18.1 [16.1; 20.2]	47.9 [38.8; 57.0]
	Hydraulic variables	28.8 [27.0; 30.6]	47.5 [42.6; 52.4]
**Thermal regime**	Air temperature	22.2 [20.0; 24.2]	46.2 [37.9; 54.5]
	Water temperature	31.2 [29.0; 33.4]	53.9 [46.9; 60.9]
**Surrogate variables**	Air temperature + discharge	37.8 [35.6; 40.0]	50.7 [45.2; 56.2]
**Direct variables**	Water temperature + hydraulic variables	40.8 [38.4; 43.2]	54.7 [48.6; 60.8]

**Table C** *Common*	**Environmental variables**	**Explanatory R^2**	**Predictive R^2**
**Hydrological regime**	Discharge	26.3 [22.9; 29.7]	61.7 [47.4; 74.4]
	Hydraulic variables	43.7 [40.7; 46.6]	61.3 [53.7; 68.9]
**Thermal regime**	Air temperature	33.6 [30.2; 37.1]	49.4 [39.8; 59.0]
	Water temperature	48.4 [44.7; 52.0]	63.7 [54.8; 72.6]
**Surrogate variables**	Air temperature + discharge	52.3 [49.0; 55.5]	61.6 [54.7; 68.5]
**Direct variables**	Water temperature + hydraulic variables	57.5 [54.3; 60.8]	67.8 [61.2; 74.4]

Sensitivity analysis of the results from RDA to uncertainties in environmental variables derived from physical-based models

In the manuscript, we used environmental variables derived from physical-based models with known uncertainties. In order to assess the influence of these uncertainties on the results from RDA, we performed a sensitivity analysis as follows. We used the literature values for uncertainties on surrogate and hydraulic variables. Biases of 0.3m, 0.3m.s^{-1} and 1°C were used for water depth and width, current velocity and air temperature, respectively [1,2]. For water temperature, a bias of 0.8°C was considered, corresponding to the standard deviation of the errors between observed and predicted annual averages (Fig 1.A; [3]). For discharge, a relative bias of 10.6% was considered, according to the standard deviation of the errors between observed and predicted annual averages (Fig 1.B; [3]). As we assume the error to be zero-centered (as shown on Fig. 1 for water temperature and discharge), the previously specified biases associated to each modelled variable was used as standard deviation of a normal distribution centered at 0 to randomly sample errors.

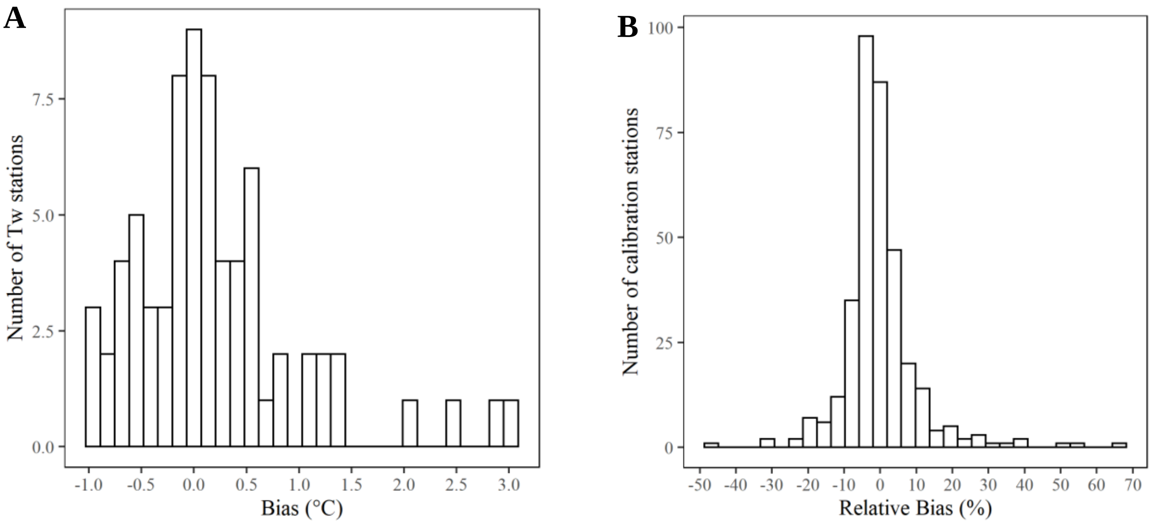

Fig 1. Distribution of the annual biases between observed and simulated values for water temperature (A) and discharge (B) provided by TNET and EROS modelling, respectively. Adapted from Seyedhashemi et al. 2022 [3].

To perform this sensitivity analysis, we replicated the cross-validation procedure previously described (see main text for details), except that each value of water temperature, air temperature, discharge and hydraulic variables was modified by adding a randomly sampled error obtained as described above (i.e. normal distribution centered at 0 with standard deviation equal to the bias of the model of the considered parameter). The cross-validation procedure involved repeating the analysis 999 times with a random sub-sampling of 70% of the sub-basins.

It should be noted that we used multidecadal interannual average values (calculated on the 1990-2010 period) of the environmental variables derived from physical-based models in the analyses presented in the main text. Mechanistically, the biases considered in the sensitivity analysis that were derived from annual averages are higher than the biases associated to multidecadal interannual averages. Therefore, the degree of sensitivity of our results to

environmental model uncertainties as assessed by this sensitivity analysis is likely to be overestimated compared to the actual sensitivity associated with the use of interannual averages.

Results of the sensitivity analysis for each of the three biological groups are presented in Table 2. For all three biological groups, the difference in R^2 between each pair of direct and surrogate variables (i.e. water temperature vs. air temperature, hydraulic variables vs. discharge, and direct vs. surrogate combined) remained statistically significant when uncertainties in the environmental variables derived from physical-based models were accounted for, as shown by the non-overlapping confidence intervals. For a few comparisons (hydraulic variables vs discharge for macroinvertebrate and common datasets, direct vs surrogate variables combined for macroinvertebrate dataset), differences were statistically significant but only marginally (i.e. overlapping confidence intervals). In all cases, the direct variables explained all three biological ordinations better than the surrogate variables.

Table 2. Sensitivity analysis of the results from RDA to uncertainties in environmental variables derived from physical-based models. Importance of the different environmental variables in explaining the biological ordination of fish, macroinvertebrate and common datasets: explanatory R^2 and associated 95% confidence intervals was derived from RDA performed on reaches contained in 70% of sub-basins. Environmental data were affected by a random sampling of errors in a normal distribution centered at zero and with the uncertainties of environmental models as standard deviation.

		**Biological datasets**		
	**Environmental variables**	**Fish**	**Macroinvertebrate**	**Common**
**Hydrological regime**	Discharge	28.4 [25.7 ; 31.1]	18.5 [16.5 ; 20.5]	27.1 [23.7 ; 30.5]
	Hydraulic variables	35.7 [33.1 ; 38.3]	21.6 [19.7 ; 23.5]	33.2 [29.8 ; 36.6]
**Thermal regime**	Air temperature	34.1 [30.8 ;37.4]	20.0 [17.8 ; 22.2]	29.0 [25.3 ; 32.7]
	Water temperature	44.2 [41.0 ; 47.4]	29.5 [27.4 ; 31.6]	46.8 [43.2 ; 50.4]
**Others**	Slope + distance to estuary	43.4 [40.4 ; 46.4]	22.3 [20.2 ; 24.4]	37.1 [33.0 ; 41.2]
**Surrogate variables**	Air temperature + discharge	47.9 [45.3 ; 50.5]	36.5 [34.2 ; 38.8]	49.9 [46.4 ; 53.4]
**Direct variables**	Water temperature + hydraulic variables	53.8 [50.8 ; 56.8]	39.5 [37.2 ; 41.8]	57.6 [54.4 ; 60.8]

References

1. Quintana-Segui P, Le Moigne P, Durand Y, Martin E, Habets F, Baillon M, et al. Analysis of near-surface atmospheric variables: Validation of the SAFRAN analysis over France. Journal of applied meteorology and climatology. 2008;47: 92–107. doi: 10.1175/2007JAMC1636.1

2. Morel M, Booker DJ, Gob F, Lamouroux N. Intercontinental predictions of river hydraulic geometry from catchment physical characteristics. Journal of Hydrology. 2020; 124292. doi:10.1016/j.jhydrol.2019.124292

3. Seyedhashemi H., Vidal J.-P., Diamond J.S., Thiéry D., Monteil C., Hendrickx F., Maire A., Moatar F. (2022) Regional, multi-decadal analysis reveals that stream temperature increases faster than air temperature. *Hydrology and Earth System Sciences Discussions*, preprint. doi: 10.5194/hess-2021-450.
